# Ancient but dynamic: structural expansion, massive gene duplication, and transposable element colonization in a supergene controlling ant social organization

**DOI:** 10.1093/molbev/msag127

**Published:** 2026-05-27

**Authors:** Hélène Boulain, Riddhi Deshmukh, Amaury Avril, Pierre Blacher, Massimo Bourquin, Sze Huei Yek, Sacha Zahnd, Patrick Tran Van, Michel Chapuisat

**Affiliations:** Department of Ecology and Evolution, University of Lausanne, Lausanne 1015, Switzerland; Department of Ecology and Evolution, University of Lausanne, Lausanne 1015, Switzerland; Department of Ecology and Evolution, University of Lausanne, Lausanne 1015, Switzerland; Department of Ecology and Evolution, University of Lausanne, Lausanne 1015, Switzerland; Department of Ecology and Evolution, University of Lausanne, Lausanne 1015, Switzerland; Department of Ecology and Evolution, University of Lausanne, Lausanne 1015, Switzerland; Department of Ecology and Evolution, University of Lausanne, Lausanne 1015, Switzerland; Department of Ecology and Evolution, University of Lausanne, Lausanne 1015, Switzerland; Department of Ecology and Evolution, University of Lausanne, Lausanne 1015, Switzerland

## Abstract

Supergenes are clusters of linked loci that underlie complex alternative phenotypes, such as colony social organization in ants. In many species of the genus *Formica*, a 30 million-year-old supergene determines whether colonies have one queen (monogyny) or multiple queens (polygyny), yet the detailed architecture of this genetic polymorphism remains poorly known. Here, we investigate the structural and functional evolution of the supergene haplotypes controlling alternative social forms in *Formica selysi*. The comparison of chromosomal-level genome assemblies for each social form reveals a 13.8-Mbp long rearranged supergene comprising three large inversions and a transposition, resulting in reduced recombination and high differentiation between haplotypes. The rearranged, derived polygynous haplotype has accumulated transposable elements (TEs) and gene duplicates. It also exhibits haplotype-specific gene expression and gene specialization. Notably, the *Formica* genus shows a large expansion of the Ubiquitin Conjugation Factor E4 B gene family, which is significantly enriched in the supergene. Despite its ancient origin, the supergene shows sparse signs of degeneration and little accumulation of deleterious variations. Overall, our results demonstrate that the supergene haplotype associated with multi-queen colonies has undergone enrichment of lineage-specialized single- and multi-copy genes with haplotype-specific expression patterns that likely contribute to the phenotype. A combination of relaxed and purifying selection allowed gene duplicates and TEs to accumulate, but prevented the accumulation of deleterious mutations, which helps to explain the long-term persistence of this large social supergene.

## Introduction

Alternative morphs in nature are frequently associated with large-scale structural changes, including inversions, translocations, and chromosomal fusions (Wellenreuther et al. 2019; Mérot et al. 2020). Such rearranged genomic elements can form a “supergene architecture” that links genes and alleles into a novel haplotype restricted to one morph and showing greatly reduced recombination with the ancestral haplotype (Schwander et al. 2014; Thompson and Jiggins 2014; Charlesworth 2016; Gutiérrez-Valencia et al. 2021). Supergenes often persist as balanced polymorphisms due to heterozygote advantage, disassortative mating or other mechanisms of negative frequency-dependent selection (Llaurens et al. 2017). Widely studied examples include the self-incompatible pin and thrum floral types in *Primula* and *Linum* (Li et al. 2016; Gutiérrez-Valencia et al. 2022), mating morphs in ruffs (Küpper et al. 2016; Lamichhaney et al. 2016; Loveland et al. 2025) and white-throated sparrows (Merritt et al. 2020; Jeong et al. 2022), migratory behavior in fishes (Pearse et al. 2019; Matschiner et al. 2022; Jamsandekar et al. 2024), mimetic wing pattern polymorphism in *Heliconius* butterflies (Joron et al. 2006; Jay et al. 2018), and social organization (colony queen number) in *Solenopsis* (Wang et al. 2013; Yan et al. 2020) and *Formica* ants (Purcell et al. 2014; Brelsford et al. 2020). These supergenes vary in size, age, complexity, and evolutionary trajectories. Few supergenes have been well characterized, and the structure, function, and evolution of most ancient, large autosomal supergenes remain poorly understood.

The suppression of recombination associated with structural variation has profound and contrasted consequences on the fate of supergenes. First, suppressed recombination can link co-adapted alleles that are beneficial in one of the morphs, and prevent maladaptive combinations (Schwander et al. 2014; Thompson and Jiggins 2014; Gutiérrez-Valencia et al. 2021; Berdan et al. 2023). Over time, additional genes that increase the fitness of that morph can be recruited into the nonrecombining region, leading to expansion and gene enrichment of the supergene (Gutiérrez-Valencia et al. 2021). Second, regions of low recombination can accumulate selfish genetic elements such as transposable elements (TEs) and transmission ratio distorters (Kent et al. 2017; Chapuisat 2023). In many cases, recombination between ancestral and derived haplotypes is reduced to such an extent that genetic exchange can only occur through double crossovers or gene conversion (Korunes and Noor 2019). The absence of recombination makes supergenes prone to accumulate deleterious mutations, leading to mutation load, recessive lethality and eventually gene loss (Küpper et al. 2016; Tuttle et al. 2016; Stolle et al. 2019; Jay et al. 2021). Complex selective pressures and variable recombination rates can act on different elements of a supergene (e.g. adjacent inversions), influencing its genomic evolution and long-term maintenance.

Insights into the long-term evolution of supergenes can be gained by comparisons with sex chromosomes (Charlesworth 2016). Sexual dimorphism is partly encoded by physically linked genes on sex chromosomes that are inherited together with little to no recombination (Saunders and Muyle 2024). We can thus consider sex chromosomes as large, ancient supergenes that evolved from autosomes (Charlesworth 2016). Gradual structural and functional divergence between two proto sex chromosomes often forms distinct evolutionary strata that reflect the expansion of the nonrecombining regions through successive steps of recombination arrest (Charlesworth 2008; Filatov 2022). Over time, the nonrecombining chromosome in the heterogametic sex typically accumulates deleterious mutations, loses genes, and eventually degenerates and shrinks (Charlesworth and Charlesworth 2000; Bachtrog 2013; Saunders and Muyle 2024). To what degree this canonical model of sex chromosome evolution applies to autosomal supergenes remains an open question. Supergenes that develop recessive lethality share properties of Y- and W-like sex chromosomes, due to their hemizygous nature and lack of recombination (e.g. Tuttle et al. 2016), yet some of them can expand and accumulate repetitive elements (e.g. Stolle et al. 2019; Jay et al. 2021). It remains unclear whether the evolutionary trajectories of supergenes inevitably lead them to shrink and degenerate, and whether this degeneration is age-dependent.

One of the oldest known autosomal supergenes occurs in the *Formica* genus and is associated with social polymorphism. The Alpine silver ant, *Formica selysi*, therefore provides an excellent system to investigate the evolution of a large, ancient supergene. This species has two types of colonies, single-queen (monogynous) colonies and multi-queen (polygynous) colonies (Chapuisat et al. 2004). The monogynous and polygynous social forms differ in several morphological, physiological and behavioral traits including dispersal, fecundity, and propensity to fuse and become multi-queened (Fontcuberta et al. 2021; Blacher et al. 2023; De Gasperin et al. 2024, 2025). The social polymorphism is controlled by a large supergene spanning most of chromosome 3, with two nonrecombining haplotypes, *M* and *P* (Purcell et al. 2014). In *F. selysi,* all individuals of monogynous colonies exclusively carry the ancestral *M* haplotype, while all individuals in polygynous colonies carry at least one copy of the derived *P* haplotype (Purcell et al. 2014; Avril et al. 2019). The *P* haplotype selfishly distorts transmission ratio, so that neither *MM* females nor *M* males are produced in polygynous colonies (males are haploid in ants; Avril et al. 2020). *PP* individuals are common in polygynous colonies, but the *P* haplotype carries a cryptic recessive load: *PP* females have reduced survival and fecundity, compared to *MP* and *MM* females, and *P* males produce fewer sperm cells compared to *M* males (Blacher et al. 2023; De Gasperin et al. 2024). Explaining the evolutionary stability of the polymorphism is challenging, because the drive by the selfish *P* haplotype tends to destabilize the polymorphism (Tafreshi et al. 2022). Yet, the social supergene and its associated social polymorphism have persisted for millions of years.

Comparative genomic analyses demonstrated that the social supergene is common to many species of the genus *Formica* and has been maintained in balanced polymorphism across multiple speciation events (Brelsford et al. 2020; Purcell et al. 2021). Phylogenetic reconstruction indicates that the supergene is basal to the genus, originating between 20 and 40 Mya (Brelsford et al. 2020), likely after its split from the *Iberoformica* lineage. Genome comparisons revealed that a small set of key trans-specific SNPs have been conserved across species, even among obligately polygynous *Formica* that have secondarily lost the rearranged *P* haplotype (Sigeman et al. 2025), while rare recombination events have homogenized other regions along the entire supergene (Brelsford et al. 2020; Purcell et al. 2021). As a result, the ancient supergene appears to follow an “eroded strata model”, where key genes have been retained by selection. Based on previous evidence, the supergene was thought to have four inversions (Brelsford et al. 2020). However, the detailed gene content of each haplotype and location of the breakpoints have not been investigated so far. Moreover, while the standard model for ancient sex chromosomes predicts deleterious mutation accumulation and degeneration, we do not yet know the extent to which these processes have occurred on the inverted, derived haplotype, which could recombine in homozygous females.

Here, we present a high-quality chromosome-scale genome assembly for each social form of *F. selysi* and infer the structure and evolution of the supergene haplotypes underpinning alternative social organization. We show that the derived, polygyny-associated, *P* supergene haplotype consists of three large inversions and a within-chromosome transposition. The *P* haplotype accumulated TEs, gene duplicates, several lineage-specific multi-copy genes, and an extraordinary expansion of the *Ubiquitin Conjugation Factor E4 B* (*UBE4B*) genes. Additionally, we investigated whether the two supergene haplotypes differed in sequence, expression, signatures of selection, and recombination rates. Lastly, contrary to our expectation, we found that the *P* supergene haplotype shows little to no deleterious accumulation of mutations in the coding regions of its genes, despite its ancient origin and cryptic load. Together, our results showing the genetic complexity underpinning the multi-faceted alternative social phenotypes, coupled with the paucity of signs of negative selection, deviate from current evolutionary models for old supergenes and sex chromosomes.

## Results

### Chromosome-scale assemblies of *F. selysi* genome

We generated high quality chromosome-scale genome assemblies for the monogynous (M) and the polygynous (P) social forms of *F. selysi* using a combination of whole-genome sequence data from PacBio long-read sequencing and Illumina short-read sequencing, Hi-C chromosome conformation capture, and high-density linkage maps (Table 1; Table S1; Figure S1). The genome of the P social form is 276.1 Mbp in length, compared to 273.1 Mbp for the M social form, a difference of ∼3 Mbp, which is supported by genome size estimates from individually re-sequenced workers (Figure S2). For each social form, more than 97% of the sequences were assembled within 27 scaffolds, the haploid number of chromosomes reported for *F. selysi* (Hauschteck-Jungen and Jungen 1976). Estimates of genome completeness, were 98% for the M and 97.7% for the P assembly, based on the BUSCO conserved Hymenoptera single-copy orthologs dataset (Table 1). We annotated protein-coding genes using RNA-sequencing data from workers, virgin and mated queens (whole-body minus gaster of both *MM* and *PP* genotypes). The *F. selysi* genome was predicted to contain 11,201 unique protein-coding genes, of which 10,774 (96%) were common to both assemblies, 94 (1%) were restricted to the M assembly, and 333 (3%) were only found in the P assembly (Table 1, Figure S3). These assemblies are contiguous, complete, accurate, and of similar quality to chromosome-scale assemblies of other ant genomes (McKenzie and Kronauer 2018; Shields et al. 2018; Gao et al. 2020; Vizueta et al. 2025).

**Table 1:** Genome assembly and annotation statistics for the monogynous (M) and polygynous (P) social forms of *Formica selysi*.

Genome	*F. selysi* M	*F. selysi* P
Assembly	FsiM_PB_v5	FsiP_PB_v5
Sequencing approach	PacBio + HiC + Illumina	PacBio + HiC + Illumina
Base pairs (Mbp)	273.1 (265.8 in scaffolds)	276.2 (268.2 in scaffolds)
% of Ns	0.05	0.06
# of chromosomes (scaffolds)	27	27
N50 chromosomes (Mbp)	10.69	10.66
# of contigs	516	544
N50 contigs (Mbp)	0.31	0.38
% of assembly in chromosomes	97.3	97.1
GC content (%)	36.17	36.09
Protein-coding genes	10,868	11,107
Transcripts	11,952	12,242
BUSCO^a^	C:98.6% [S:98.0%,D:0.6%],F:0.7%,M:0.7%	C:98.4% [S:97.7%,D:0.7%],F:0.7%,M:0.9%
Transposable elements (%)^b^	29.2	29.8

^a^C: complete, S: single-copy, D: duplicated, F: fragmented, M: missing, based on a set of 5,991 single-copy genes.

^b^% of genome occupied by transposable elements.

### The *P* haplotype is rearranged and larger

**Figure 1:**
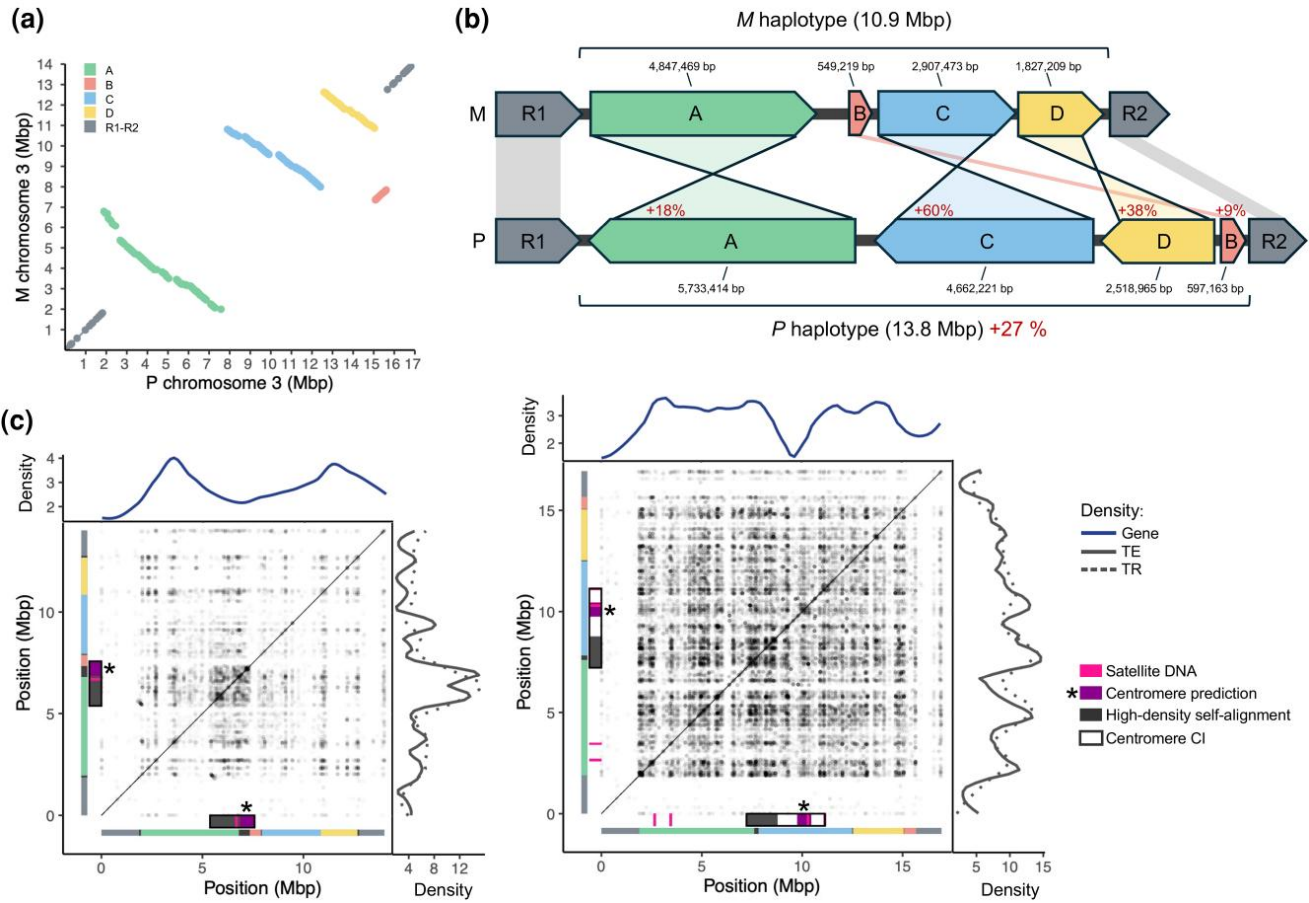
The “polygynous” *P* haplotype is rearranged, with three large inversions and a transposition. The *P* haplotype is also 27% larger than the “monogynous” *M* haplotype and shows different centromere location. (a) Dotplot of chromosome 3 alignment between the M and P assemblies. The social supergene consists of three large inversions (regions A, C and D) and a within-chromosome transposition (region B). The supergene is flanked by colinear extremities of chromosome 3 (R1 and R2). (b) Structure and size differences between the *M* and *P* supergene haplotypes. Percentages in red indicate size increases of the *P* haplotype compared to the *M* haplotype. (c) Prediction of chromosome 3 centromere location. Dotplots show self-versus-self alignment of chromosome 3 (from M and P assemblies in the left and right panels, respectively) with alignment length > 100 bp. The more repetitive a region is, the more it self-aligns and appears darker on the dotplot. Centromere confidence intervals (CI, black box) encompass (i) regions with high density of self-alignment appearing in grey (calculated over 100-kb windows), (ii) predicted centromere range shown in violet and (iii) satellite DNA matches in pink. Top plots indicate gene density, while right plots indicate transposable elements (TE) and tandem repeats (TR) densities. The lines show LOESS smoothed averages of 50-kb fixed windows. Chromosome 3 centromere lies in the supergene. The chromosome 3 carrying the *M* haplotype is metacentric, while the chromosome 3 carrying the *P* haplotype is submetacentric.

The haplotype-resolved genome assemblies for the monogynous and polygynous social forms of *F. selysi* reveal structural variations on chromosome 3, consistent with previous findings on this social supergene (Purcell et al. 2014; Brelsford et al. 2020). Pairwise alignment of chromosome 3 from the M and P assemblies reveals a large, rearranged region in the center of the chromosome, flanked by two synteny blocks at the extremities (Fig. 1a). The extensive genomic rearrangements within the *P* haplotype result from three large genomic inversions (regions A, C, D) that also produced a within-chromosome transposition (region B; Fig. 1a). Overall, the derived *P* haplotype (13,805,174 bp) is 27% larger than the ancestral *M* haplotype (10,893,292 bp) and amounts to 81.5% of the entire chromosome. Levels of genomic expansion differ significantly across inversions, with regions A, B, C and D being 18, 9, 60 and 38% larger in the *P* haplotype, compared to the *M* haplotype (Fig. 1b). In contrast, the breakpoint regions (nonaligning regions between inversions) are 2.6 times larger in the *M* haplotype (761,911 bp), compared to the *P* haplotype (293,400 bp). The flanking regions (R1–R2) are collinear and of similar length, amounting to a cumulative size of 3.10 Mbp (Fig. 1a). Due to the genomic rearrangements and expansions of the *P* haplotype, the predicted centromeres localize in different areas of chromosome 3 for each haplotype (Fig. 1c). In chromosome 3 carrying the *M* haplotype, the centromere is metacentric and spans 2.2 Mbp between regions A and B of the supergene. In chromosome 3 carrying the *P* haplotype, the centromere is located primarily within region C of the supergene and spans 3.9 Mbp, in a submetacentric position. Both predicted centromeres lie in regions with high densities of TEs and tandem repeats (TRs), and low gene density (Fig. 1c).

### Low recombination and high genetic divergence between supergene haplotypes

**Figure 2:**
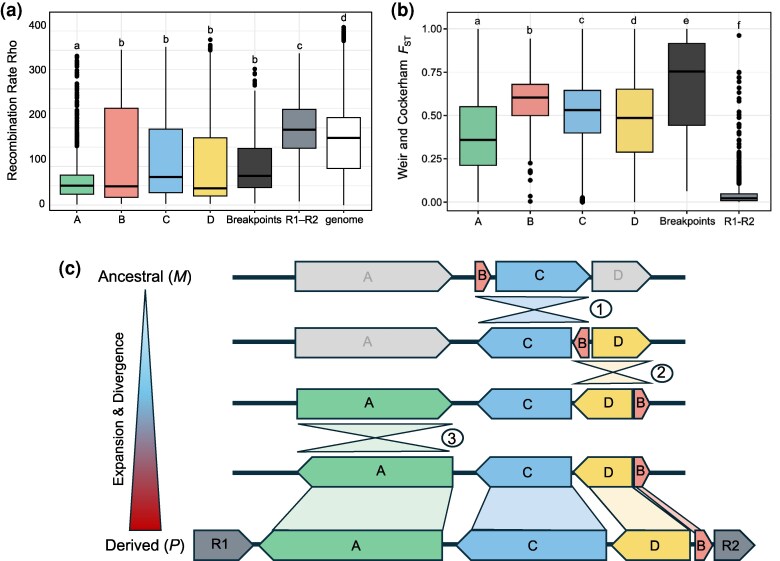
Reduced recombination rate and high genetic divergence between regions of chromosome 3 reveal how subsequent inversions gave rise to the *P* supergene haplotype. (a) Recombination rates across chromosome 3 regions and the rest of the genome, estimated for workers from monogynous and polygynous colonies (*MM*, n = 21 colonies from four populations; *MP*, n = 12 colonies from four populations; *PP*, n = 15 colonies from two populations) using 100-kb sliding windows with a 20-kb step. All regions of the supergene show lower recombination rates than R1–R2 region and the rest of the genome (*χ*^2^ = 1,366.2, df = 6, *P* < 0.001). Letters refer to Dunn's post hoc test comparison with Bonferroni correction after a Kruskal–Wallis. (b) Genetic divergence between monogynous and polygynous social forms calculated using weighted Weir and Cockerham *F*_ST_ estimates in 1-kb windows across regions of chromosome 3. Letters indicate with post hoc Wilcoxon rank sum test with Bonferroni correction after a Kruskal–Wallis (*χ^2^* = 4,815.6, df = 5, *P* < 0.001). (c) Evolutionary scenario (based on genetic divergence and expansion of rearranged regions in the *P* haplotype) for the sequence of inversions leading to the current architecture of the supergene with three inversions and a transposition within the same chromosome resulting from the first two sequential inversions. Numbers indicate the probable order of inversion events.

Chromosomal inversions often lead to reduced recombination between ancestral and rearranged haplotypes. At the population level (individuals of all genotypes), every region of the supergene shows a reduced rate of recombination compared to both collinear regions flanking the supergene (R1–R2) and the rest of *F. selysi* genome (Fig. 2a). These recombination rates seem consistent across regions, except in A, which encompasses some of the centromeric region in the *M* haplotype (Fig. 1c) and thus shows an even lower recombination rate (Fig. 2a, Figure S4). At the level of individual genotypes, surprisingly, a pattern of strongly reduced recombination at the supergene is not only observed for *MP* but also for *PP* individuals, while *MM* individuals show similar recombination rates at the supergene and the rest of the genome (Figure S4). Reduced rates of recombination within the supergene in polygynous individuals are likely due to inversions for *MP* individuals and to lower genetic diversity for *PP* individuals, which came from two populations instead of four (Figure S4c). The supergene haplotypes show high genetic divergence (*F*_ST_) between social forms across all regions of the supergene, but not in the shared collinear regions flanking the supergene (R1–R2) (Fig. 2b). The level of genetic divergence is highest in breakpoints, and decreases gradually in regions B, C, D, and A. Based on the patterns of genetic divergence (Fig. 2a) and expansion of the *P* haplotype (Fig. 1b), we propose a parsimonious scenario involving a sequence of three inversions that gave rise to the current chromosomal architecture of the *P* haplotype (Fig. 2c).

### The *P* haplotype exhibits increased and active accumulation of TEs

**Figure 3:**
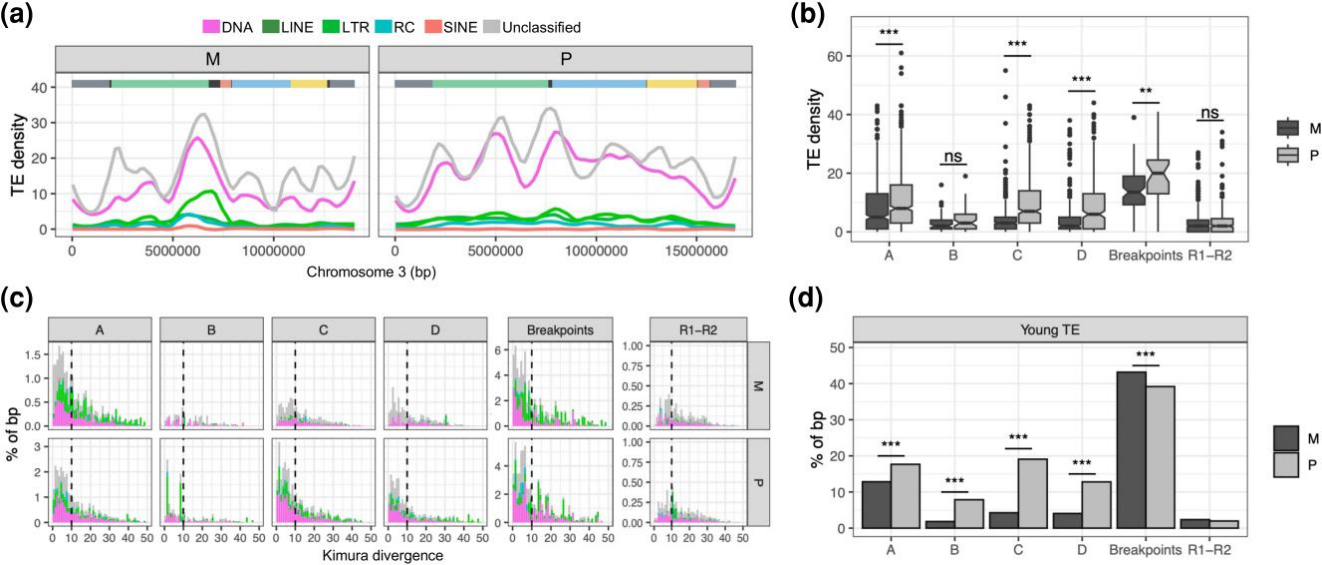
Increased and region-specific accumulation of young transposable elements in the *P* haplotype. (a) Density of transposable elements (TEs) along chromosome 3 from “monogynous” M and “polygynous” P genome assemblies. DNA, DNA transposons; LINE, long interspersed nuclear element; LTR, long terminal repeat retrotransposons; RC, rolling-circle transposons; SINE, short interspersed nuclear elements. Lines represent LOESS smoothed averages over 50-kb fixed windows. Supergene regions are color coded as in Fig. 1. (b) TE densities in 10-kb fixed for each supergene region. Regions A (*W* = 120,022, *P* = 0.000107), C (*W* = 39,110, *P* < 6.11e-23), D (*W* = 17,172, *P* = 0.00000478), and breakpoints (*W* = 928, *P* = 0.00254) show significantly higher TE density in the *P* haplotype. (c) TE age landscapes (measured as Kimura divergence from consensus sequences) within the different regions of M and P chromosomes 3. Colors refer to TE classes as shown in (a). The dotted black line marks 10% divergence from consensus, below which TEs are considered young. (d) proportion of genomic span occupied by young TEs in different regions of *M* and *P* haplotypes. Proportion tests indicate a significantly higher percentage of young TEs in most *P* haplotype regions. Statistical significance: ***p < 0.001, **P < 0.01.

TEs account for about 29% of the *F. selysi* genome (Table 1), but their distribution is highly heterogeneous across chromosomes (ranging from 2 to 50%, Figure S5). The *P* haplotype of the supergene contains 3.8 Mb of TEs (27.5% of its length), compared to 2.02 Mb (18.5%) in the *M* haplotype, representing 1.88-fold more TE sequence overall (*χ*^2^ = 271,239, df = 1, *P* < 0.001). As in the rest of the genome, most supergene TEs belong to class II DNA transposons and unclassified repeats, with a notable local enrichment of class I long terminal repeat retrotransposons (LTR) at the predicted chromosome 3 centromere in the *M* haplotype (Fig. 3a). All regions of the supergene, except region B, show significantly higher TE density in the *P* haplotype (Fig. 3b), with the strongest enrichment in region C, which corresponds to the oldest inversion and contains 2.14 times more TEs in *P* than in *M*. TE age profiles reveal that this excess is driven by a recent burst of TE insertions in the *P* haplotype, which shows a pronounced peak of young TEs (<10% divergence) in supergene regions A, B, C and D whereas this pattern is only observed in region A for the *M* haplotype (Fig. 3c). Consequently, young TEs span a greater proportion of regions A, B, C, D in the *P* haplotype than in the corresponding *M* regions (*χ*^2^ tests, all *P* < 2.2 × 10^−16^; Fig. 3d). This recent invasion suggests that *P* haplotype expansion is not solely due to long-term accumulation under reduced recombination, but also reflects ongoing mobilization of multiple TE classes, potentially facilitated by relaxed purifying selection, and/or changes in TE regulation. In absolute terms, the breakpoint regions of both haplotypes are heavily colonized by TEs, which cover 0.76 Mb (70%) in *M* and 0.29 Mb (68%) in *P.* This TE accumulation is ongoing, with young TEs spanning 43% and 39% of the breakpoint regions in *M* and *P*, respectively. As expected for recombining regions, TEs are not enriched in the collinear regions flanking the supergene (R1–R2), which show low TE densities and low proportions of young TEs (Figs 3b–d).

### Limited evidence for degeneration and directional selection in the gene-rich supergene

**Figure 4:**
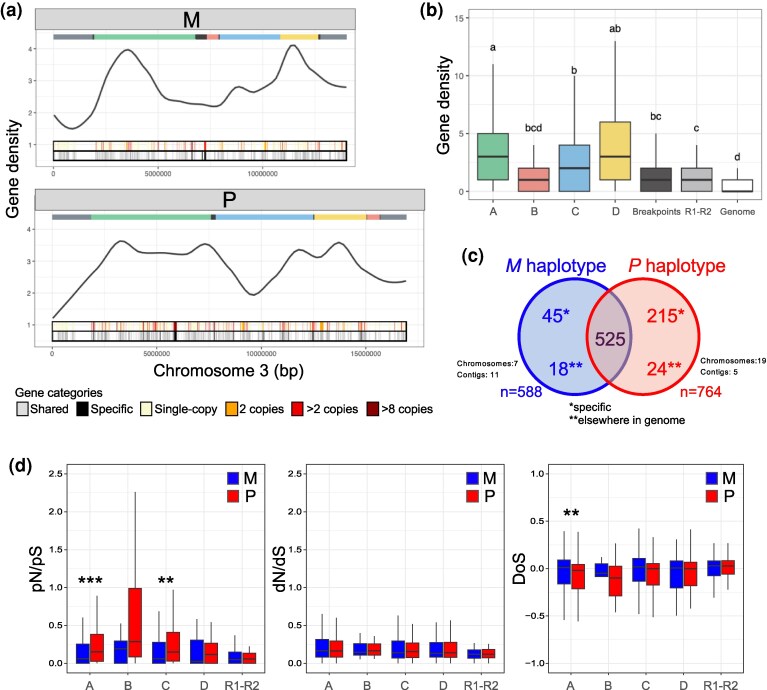
Higher gene density within *F. selysi* supergene. (a) Gene density across chromosome 3 from “monogynous” M (top panel) and “polygynous” P genome assemblies (bottom panel). Lines show LOESS smoothed averages of number of genes per 50-kb fixed windows. Supergene regions are indicated by colors as defined in Fig. 1. Maps at the bottom of each plot indicate (i) genes shared by the two assemblies (shared) versus genes uniquely annotated in one assembly (specific), and (ii) single-copy genes vs genes from multigenic families. (b) Comparison of gene densities over 50-kb fixed windows between the different regions of the chromosome 3 and the rest of the genome. Windows from each assembly were pooled together as there is no significant difference between *M* and *P* haplotypes (Figure S6). Every region of the supergene, except region B, shows higher gene density than the rest of the genome (*χ*^2^ = 704.94, df = 6, *P* < 0.001). Letters refer to Dunn's post hoc test comparison with Bonferroni correction. (c) Gene overlap between the 588 and 764 genes from the *M* and *P* haplotypes of the supergene, respectively. ** represents genes found in one haplotype of the supergene while being located on contigs or chromosomes within the other haplotype. (d) Distributions of ratios of nonsynonymous to synonymous substitutions within *F. selysi* (pN/pS), between *F. selysi* and *Polyergus* species (dN/dS) and direction of selection (DoS) for each region of chromosome 3. Statistical comparisons were made using the Wilcoxon rank sum test, **P < 0.01, ***P < 0.001.

Gene density varies across chromosome 3 in both M and P genome assemblies, with notable enrichment in the supergene compared to the collinear regions R1–R2. Peaks in gene density are observed in regions A and D for both haplotypes, with a drop near the centromere (Fig. 4a). Mapping of gene content reveals a high proportion of haplotype-specific genes within the *P* haplotype of the supergene, primarily concentrated in regions A, C, and D (Fig. 4a). Further analysis confirms significantly higher gene density in the supergene regions A, C, D, and breakpoints, compared to the rest of the genome (Fig. 4b). Gene density does not significantly differ between the *M* and *P* haplotypes, but the *P* haplotype is larger and has accumulated more genes (Figure S6). In total, the supergene harbors 827 genes, with 588 present in the *M* haplotype and 764 in the *P* haplotype. Out of these 827 genes, 525 (63%) are shared between the two haplotypes, while 63 (7%) are specific to *M* and 239 (30%) are specific to *P*. Note that 18 of the *M*-specific genes and 24 of the *P*-specific genes are located on different chromosomes or contigs in the assembly of the other social form, suggesting occasional discrepancies in the two independent assemblies (Fig. 4c). The remaining 45 *M*-specific and 215 *P*-specific genes are unique to their respective haplotypes. Comparing the ratios of synonymous and nonsynonymous changes, and the direction of selection (DoS) in protein-coding genes shared between the *M* and *P* haplotypes, reveals only minimal difference across the supergene (Fig. 4d, Figure S7), except for region A, which shows decreased DoS in the *P* haplotype (Fig. 4d). This is attributed to slightly higher rates of polymorphism (pN/pS) (significant only in A and C) and similar rates of dN/dS in regions A–D of the supergene in the P assembly compared to the M assembly and the rest of the genome (Figure S7). Therefore, among genes shared between the two assemblies, the supergene is not enriched in nonsynonymous substitutions and shows no clear evidence of deleterious mutation accumulation across rearranged regions of the *P* haplotype.

### The *P* haplotype is enriched in recently duplicated genes

**Figure 5:**
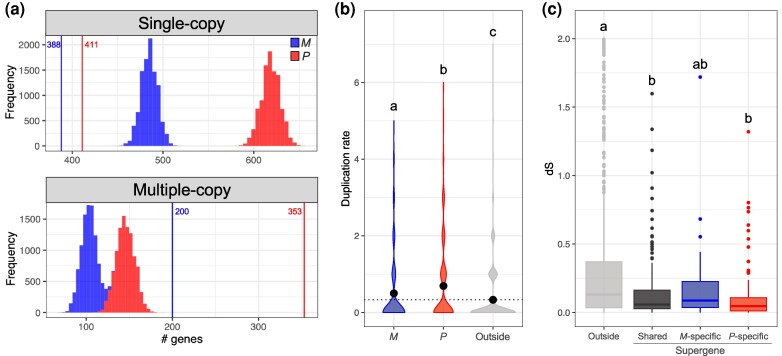
Enrichment and recent origin of duplicated genes in the supergene haplotypes. (a) Observed versus simulated counts of single-copy (top panel) and multiple-copy (bottom panel) genes in the “monogynous” (*M*) and “polygynous” (*P*) supergene haplotypes. Blue and red histograms show simulated distributions based on randomly resampling 588 and 764 genes for the *M* and *P* haplotypes, respectively, from the genome-wide distribution of single-copy (top) and multiple-copy (bottom) genes in *F. selysi* (10,000 iterations). Vertical lines represent observed values. Both haplotypes show a deficit of single-copy genes (*M*: 95% confidence interval [467, 502], *P* < 0.001; *P*: 95% confidence interval [598, 638], *P* < 0.001) and an enrichment in multiple-copy genes (*M*: 95% confidence interval [86, 121], *P* < 0.001; *P*: 95% confidence interval [126, 166], *P* < 0.001). (b) Duplication rate (duplicated genes per 50-kb fixed window) in each supergene haplotype and the rest of the genome (black dots show averages). Duplication rates are significantly higher in the *P* haplotype (0.69 duplicated gene per 50-kb window) than in the *M* haplotype (0.5 per 50-kb), and both exceed the genome average (0.33 per 50-kb, dashed line). Letters denote significant differences (Dunn's post hoc test after a Kruskal–Wallis test, *χ*^2^ = 81.924, df = 2, *P* < 0.001). (c) Synonymous substitution rates (dS) for duplicated gene pairs by location. “Outside” = elsewhere in the genome; “shared” = present in both haplotypes; “*M*-specific” and “*P*-specific” = specific to each haplotype. Letters denote significant differences (Dunn's post hoc test after a Kruskal–Wallis test, *χ*^2^ = 50.932, df = 3, *P* < 0.001).

Increased gene density in the supergene region is primarily due to an accumulation of duplicated genes. Both haplotypes contain significantly more multiple-copy genes and fewer single-copy genes than expected at random across *F. selysi* genome (Fig. 5a). Within the supergene, the duplication rate is substantially higher than in the rest of the genome, with 49% of the 827 supergene genes belonging to multiple-copy families, compared to only 17% genome-wide. Both *P* and *M* haplotypes show elevated duplication rates relative to the genome average, and the *P* haplotype contains more duplicated genes than the *M* (Fig. 5b). Most haplotype-specific genes originated from duplication events, with 186 out of 215 *P*-specific genes (87%), and 40 out of 45 *M*-specific genes (89%) belonging to multiple-copy families. In addition, 32% of the genes shared between haplotypes also originated from duplications. Both shared and *P*-specific duplications have, on average, lower synonymous substitution rates (dS) than duplications elsewhere in the genome (Fig. 5c), suggesting a more recent origin. Shared duplications likely arose during the successive genomic rearrangements that suppressed recombination between haplotypes (Fig. 2c), whereas *P*-specific duplications probably originated afterward. This pattern is not observed for *M*-specific duplications, possibly because there are fewer of them.

### Lineage specialization of the supergene

**Figure 6:**
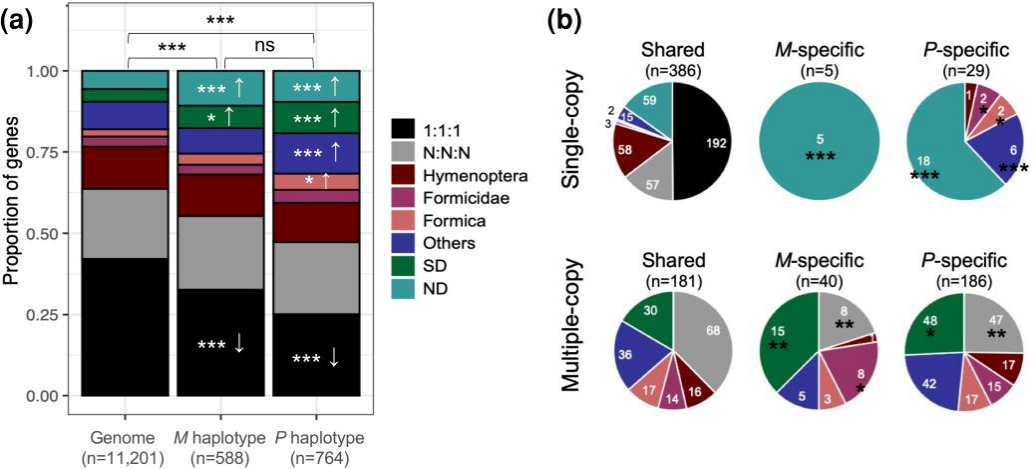
Supergene haplotypes are enriched in lineage-specific genes. (a) Proportions of orthologue categories in *F. selysi* whole genome, *M* haplotype of the supergene, and *P* haplotype of the supergene. The orthologue categories determined across 10 insect genomes are listed and color coded in the legend on the right. SD indicates species-specific duplicated genes, and ND species-specific single-copy genes. For each orthologue category, absence in one genome was allowed, except for the 1:1:1 category (for which absence in the outgroup *Drosophila melanogaster* was not allowed), and for the *Formica* category (see Figure S8a). The distribution of orthologues differs significantly between the two supergene haplotypes and the genome (average adjusted *P* values of <0.001). The depletion or enrichment (indicated by arrows) of certain orthologue categories in supergene haplotypes are compared to what is randomly expected genome-wide (level of significance indicated by stars) (Figure S8b). (b) Origin of single- and multiple-copy genes of *F. selysi* supergene. “Shared” genes are found in both haplotypes, while *M*-specific and *P*-specific genes are only found in one haplotype. Stars indicate significantly different proportions in haplotype-specific genes, compared to shared genes. Statistical significance: *P < 0.05, **P < 0.01, ***P < 0.001.

The complete gene set of *F. selysi* (11,201 genes) is consistent with gene counts reported for other Formicidae species, which range from 10,973 (*Carebara sp.*) to 16,903 (*Acromyrmex octospinosus*, Vizueta et al. 2025). A large proportion of these genes are conserved among insects, Hymenoptera, and Formicidae (Figure S8a). Interestingly, the orthology profiles differ significantly between the whole genome and the two supergene haplotypes, while the two haplotypes do not differ in their overall orthologue composition (Fig. 6a). Both haplotypes are depleted in highly conserved single-copy orthologues (the 1:1:1 category) and enriched in *F. selysi*-specific orphan genes, in both the duplicated (SD) and single-copy (ND) categories. The *P* haplotype is also enriched in *Formica*-specific genes and lineage-restricted orthologues (Fig. 6b, Figure S8b). Haplotype-specific genes show a significant enrichment in single- and multiple-copy *F. selysi*-specific genes and depletion in highly conserved genes (Fig. 6b), while in contrast genes shared between the two haplotypes exhibit a distribution of orthologue categories similar to the rest of the genome.

### Dominant expression of the *P* haplotype

**Figure 7:**
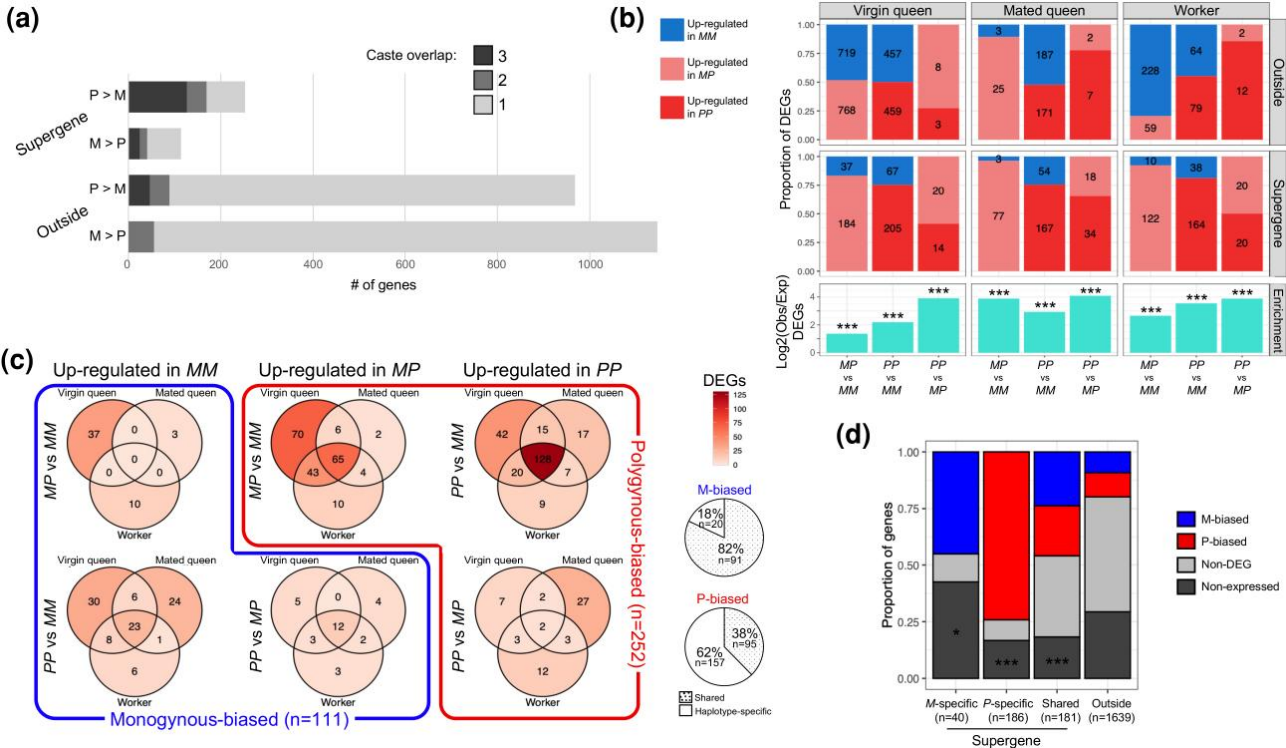
Dominant expression of the *P* haplotype across castes. (a) Overall counts of differentially expressed genes (DEGs) between *F. selysi* virgin queens, mated queens, and workers from monogynous (homozygous *MM* at the supergene) and polygynous colonies (heterozygous *MP* and homozygous *PP* at the supergene) show a bias toward polygynous (P) individuals for the supergene and toward monogynous (M) individuals for other genes. (b) DEG counts across genotypes and castes. The top panel presents the proportion of DEGs located outside of the supergene, while the middle panel shows those within the supergene. Observed DEG counts appear on the bars for each genotype comparison within the three castes. The bottom panel displays supergene enrichment of DEGs, represented as the log-transformed ratio of observed vs. expected DEGs within the supergene. A positive ratio signifies an overrepresentation of DEGs in the supergene compared to the whole genome. Stars indicate significant enrichment (*P* ≤ 0.001, χ^2^ test). (c) Euler plot overlap of genes from the supergene showing genotype-specific up-regulation in each caste. The nonredundant DEGs can be separated in two sets, a monogynous-biased set (n = 111 genes) that includes *MM* up-regulated genes and *MP* up-regulated genes (from the *MP* vs. *PP* comparison only), and a polygynous-biased set (n = 252 genes) that consists of *MP* up-regulated genes (from the *MP* vs. *MM* comparison only) and *PP* up-regulated genes. Genes with ambiguous expression or localization were excluded. Pie charts illustrate the proportions of shared and haplotype-specific genes within each set. (d) Expression categories of duplicated genes within the supergene (haplotype-specific and shared) and elsewhere in the genome (outside). Proportions of monogynous-biased, polygynous-biased, nondifferentially expressed (non-DEG), and nonexpressed genes are shown. Stars denote significant differences in the proportions of nonexpressed genes between duplicated genes inside and outside the supergene (proportion tests). Statistical significance: *P < 0.05, **P < 0.01, ***P < 0.001.

Comparisons of gene expression across the three supergene genotypes (*MM*, *MP*, and *PP*) in virgin queens, mated queens, and workers reveal stronger transcriptional shifts in individuals carrying the *P* haplotype. Although similar numbers of genes are differentially expressed between social forms (1,250 genes are up-regulated in monogynous individuals and 1,206 genes are up-regulated in polygynous individuals), fold-changes are more pronounced for genes up-regulated in polygynous individuals (polygynous-biased; Fig. 7a, Figure S9a). The supergene exhibits a clear bias toward polygynous individuals, with more than twice as many genes being up-regulated in polygynous individuals as in monogynous individuals, and about half of these differentially expressed genes (DEGs) show consistent expression patterns across all three castes. In contrast, genes up-regulated in monogynous individuals are more widely distributed across the genome and show little overlap between castes (Fig. 7a). When castes are examined separately, virgin queens show the most DEGs, followed by mated queens and workers, a pattern consistent across both supergene and genome-wide loci. Importantly, all genotype comparisons reveal a significant enrichment of DEGs within the supergene compared to genome-wide expectations. Although the supergene contains 7.4% of all genes, supergene DEGs amount to 15% of all DEGs. Moreover, most of the supergene DEGs are up-regulated in polygynous individuals, and this pattern is observed across all castes (Fig. 7b). This ubiquitous polygynous-biased expression and dominance of the *P* haplotype are also reflected in the expression profiles of heterozygous *MP* individuals, which closely resemble those of *PP* individuals, even if, compared to *PP* individuals, *MP* individuals also express genes that are up-regulated in monogynous individuals (Figure S10). The dominant effect of the *P* haplotype on transcriptional activity is further evidenced by the abundance of genes up-regulated in *MP* individuals, compared to *MM* individuals (Fig. 7b). Altogether, these results show that the *M* and *P* haplotypes display distinct expression profiles, with a general bias toward higher expression in the *P* haplotype.

Across castes, genotype-specific expression patterns within the supergene show limited overlap among *MM* up-regulated genes, but substantial overlap for *MP* and *PP* up-regulated genes (Fig. 7c). A similar pattern is observed for DEGs outside the supergene, but with a somewhat reduced overlap across castes among *MP*- and *PP*- up-regulated genes (Figure S9b). Based on these patterns, we identify two major gene sets within the supergene, one comprising 111 genes over-expressed in the monogynous social form (monogynous-biased) and another comprising 252 genes over-expressed in the polygynous social form (polygynous-biased; Fig. 7c). The dominant expression of the *P* haplotype is largely driven by haplotype-specific genes, with 62% of polygynous-biased genes found exclusively in the *P* haplotype (Fig. 7c). Given the enrichment of duplicated genes in both haplotypes, particularly in *P*, we assessed their expression and found that most duplicates within the supergene, whether shared or *P*-specific, are actively transcribed and contain proportionally fewer nonexpressed genes than duplicated genes elsewhere in the genome. In contrast, *M*-specific duplicates within the supergene show a higher proportion of nonexpressed genes (Fig. 7d).

### Expression and evolutionary patterns of established polygyny candidate genes

Recent comparative genomic analyses across *Formica* species have identified 13 candidate genes potentially involved in the regulation of polygyny, based on conserved haplotype-specific and trans-specific SNPs associated with the social supergene (Brelsford et al. 2020; Purcell et al. 2021; Sigeman et al. 2025). Because these candidates are supported by trans-species conservation and provide a robust signal of long-term association with social polymorphism, we focused on this subset of genes to investigate their expression patterns, molecular evolution, and evolutionary origins in *F. selysi*. Of these 13 candidates, eight are located within inversion D of the supergene, indicating partial enrichment in this region. Orthology analysis shows that 12 of the 13 genes are single-copy genes, with seven conserved across deep phylogenetic scales (at least at the Hymenoptera level). Differential expression analyses revealed that five of the 13 candidates exhibit polygynous-biased expression (Table 2). Patterns of divergence vary among the candidates. Some genes combine coding and regulatory differentiation, including *MRPL34*, *RPUSD4* and *AmGR10*, which show fixed amino-acid substitutions specific to the *P* haplotype and P-biased expression. In other cases, divergence is restricted to either coding sequences (*Knockout*) or gene expression (*Zasp52* and *TTLL2*). Overall, previously identified trans-species candidates are predominantly conserved single-copy genes (Table 2), whereas the supergene also contains many lineage-specific and duplicated genes that may include additional candidates.

**Table 2:** Candidate genes for polygyny in *Formica* ants: gene origin, differential expression and signatures of selection in *Formica selysi*.

						*Monogynous*		*Polygynous*		
Gene name^a^ (function)	Supergene inversion	Gene ID	Gene type	Orthologue category^b^	DEG category^c^	pN/pS	dN/dS	pN/pS	dN/dS	AA changes (M→P) and consequences^d^
***STK32B* ^1^** (serine/threonine-protein kinase 32)	A	S3A_001490	Single copy	1:1:1	Ambigous	0.116	0.080	0	0.087	Ala→Val^§^ (exon 1, within protein kinase domain)
***MRPL34* ^1^** (mitochondrial ribosomal protein L34)	A	S3A_001440	Single copy	N:N:N	P-biased	0	0.122	0	0.269	Pro→Arg ^+^*^§^ (exon 2)
***RPUSD4* ^1^** (mitochondrial RNA pseudouridine synthase)	A	S3A_001439	Single copy	Hymenoptera	P-biased	0.274	0.610	0	0.514	Deletion of exons 1 & 2 (125 aa) in P due to *Tc1-Mariner* insertion
***G9A* ^1^** (histone/lysine N-methyltransferase)	D	S3D_001989	Single copy	1:1:1	Non-DEG	1.163	0.081	0.186	0.087	Thr→Met*^§^, Ile→Thr^§^, **Thr→Arg^+§^**, Gly→Glu^+^*^§^, Ile→Val, Ala→Val^§^ (exon 1, new protein–protein interaction coiled-coil domain in P); Ser→Gly*^§^ (exon 2); Leu→Phe*^§^ (exon 6); Ala→Thr*^§^ (exon 8)
***Single-minded*** ^2^ (helix-loop-helix transcription factor)	A	S3A_001449	Single copy	N:N:N	M-biased	0.362	0.068	0.036	0.070	None
***Uncharacterized protein*** ^2^ (DNA-binding protein)	C	S3C_001843	Multiple copy	N:N:N	Non-DEG	0.259	0.076	0.363	0.081	Thr→Met*^§^ (exon 1); His→Gln*^§^ (exon 5); Thr→Ala*^§^, Asn→Asp^+^* (exon 7, within ELM2 binding domain); Ser→Asn*, Ala→Thr*^§^ (exon 12)
***zinc finger 148-like*** ^2^ (metal response element-binding transcription factor)	D	S3D_001925	Single copy	1:1:1	Non-DEG	0.428	0.185	0.268	0.160	None
***AmGR10*** ^2^ *(gustatory receptor)*	D	S3D_001926	Single copy	Others	P-biased	0	0.244	0	0.171	33 amino-acid changes in last exon (shorter in P, affects alternance of transmembrane, cytoplasmic and noncytoplasmic domains)
***tplus3b*** ^2^ (testis-specific Plus3 domain b)	D	S3D_001929	Single copy	1:1:1	Non-DEG	0	0.033	0	0.039	Ala→Val^§^ (exon 1)
***Knockout*** ^2^ (storkhead-box protein 2)	D	S3D_001955	Single copy	1:1:1	Non-DEG	0	0.045	0.156	0.061	Ser→Ile*^§^ (exon 6); Val→Met*^§^, Val→Met^§^, Ser→Phe*^§^, Ala→Val^§^, Thr→Ser* (exon 7)
***FMRFaR*** ^2^ (FMRFamide receptor)	D	S3D_001983	Single copy	1:1:1	M-biased	0	0.147	0	0.160	None
***Zasp52*** ^3^ (Z-band alternatively spliced PDZ-motif protein)	D	S3D_001891	Single copy	1:1:1	P-biased	0.020	0.115	0.0631	0.117	None (spliced variants identical in M and P)
***TTLL2*** ^3^ (tubulin polyglutamylase)	D	S3D_001892	Single copy	Hymenoptera	P-biased	0	0.127	0.101	0.128	None

Monogynous and polygynous refer to the assembly of comparison

^a^Gene names and functions as defined in *D. melanogaster* or *A. mellifera* orthologues.

^b^Categories previously defined by orthology analysis across Hymenoptera and ants. 1:1:1, Single-copy conserved orthologues, N:N:N, multiple-copy conserved orthologues.

^c^Categories defined by differential expression analysis between *MM*, *MP*, and *PP* individuals. Non-DEG indicates genes which are not differentially expressed between social forms. M-biased: monogynous-biased, P-biased: polygynous-biased. Ambiguous genes were P-biased in one caste and M-biased in another.

^d^Amino-acid changes represented in bold have the strongest consequences on protein function. Underlining shows changes in polarity, + indicates changes in charge, * shows gain/loss of functional groups (hydroxyl, amid, aromatic, imidazole groups or potential for disulfide bonds) and § represents changes in size or steric hindrance.

^1^ Brelsford et al. (2020).

^2^ Purcell et al. (2021).

^3^ Sigeman et al. (2025).

### Extensive colonization of the supergene by *UBE4B* genes

**Figure 8:**
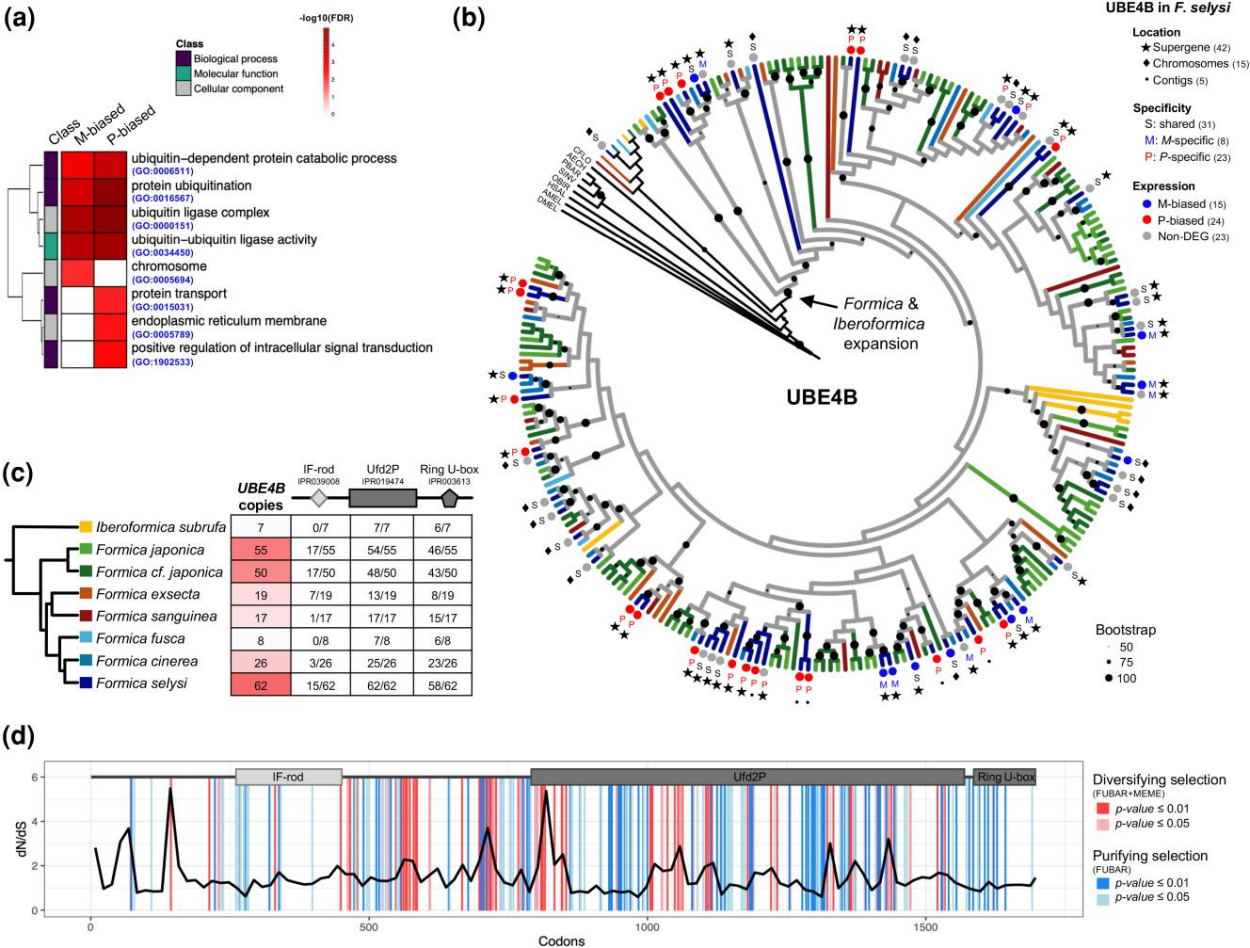
Expansion of the ubiquitin conjugation factor E4 B (*UBE4B*) gene family in the *F. selysi* supergene and in *Formica* genomes. (a) Hierarchical clustering of enriched gene ontology (GO) terms within monogynous-biased and polygynous-biased gene sets of the *F. selysi* supergene, based on false discovery rate (FDR). GO terms associated with protein ubiquitination are significantly enriched in both gene sets (b) Maximum-likelihood phylogenetic tree of UBE4B family, constructed using 252 protein sequences from seven *Formica* species, *Iberoformica subrufa*, six additional ant species, and two outgroups: *Apis mellifera* (AMEL) and *Drosophila melanogaster* (DMEL). Branch colors correspond to *Formica* species, as illustrated in panel c. Bootstrap support values (percentage of 1,000 replicates) are displayed for branches with support greater than 50%. The genomic location, haplotype specificity, and gene expression bias of the 62 *F. selysi UBE4B* genes are annotated around the tree. (c) Comparison of *UBE4B* gene counts across *Formica* and *Iberoformica* species, along with the number of proteins containing key UBE4B domains: UFd2P, Ring U-box, and the additional IF-rod domain. (d) Evolutionary rate of *UBE4B* across *Formica* species. The dN/dS ratio, averaged over 15-codon sliding windows along the UBE4B alignment, is shown. Codons under diversifying and purifying selection are highlighted in red and blue shades, respectively. Species abbreviations: AECH (*Acromyrmex echinatior*), CFLO (*Camponotus floridanus*), HSAL (*Harpegnathos saltator*), OBIR (*Ooceraea biroi*), PBAR (*Pogonomyrmex barbatus*), SINV (*Solenopsis invicta*).

Gene ontology (GO) enrichment analysis reveals a strong over-representation of ubiquitination-related processes among genes belonging to the supergene, including those showing monogynous- or polygynous-biased expression (Fig. 8a, Figure S11). This enrichment is primarily due to an expansion of the Ubiquitin Conjugation Factor E4 B (UBE4B) gene family. A total of 62 copies of *UBE4B* are identified in *F. selysi*, of which 42 (68%) are located on one or both haplotypes of the supergene (Fig. 8b, Table S2). These copies exhibit varied expression biases and haplotype specificity: 15 show monogynous-biased expression, 24 polygynous-biased expression, and 23 are not differentially expressed. At the haplotype level, eight *UBE4B* genes are specific to the *M* haplotype, 23 are specific to the *P* haplotype, and 31 are shared by both haplotypes.

Phylogenetic analysis of UBE4B proteins across species of the genus *Formica* and *Iberoformica*, six more distantly related ant species, and two outgroups (*Apis mellifera* and *Drosophila melanogaster*) indicates that the expansion of the UBE4B gene family is restricted to the genus *Formica* and its sister genus *Iberoformica* (Fig. 8b and c, Table S3). All other ant species with high-quality genome assembly available, including more than 150 species from the GAGA project (Vizueta et al. 2025), possess at most a single *UBE4B* copy. Across the diverse *Formica* species, many copies of *UBE4B* cluster with those found in the *F. selysi* supergene (Fig. 8b), suggesting that they were already present in the ancestral supergene shared by many *Formica* species (Brelsford et al. 2020; Purcell et al 2021). In contrast, all seven *UBE4B* copies in *I. subrufa* cluster with *F. selysi* copies located outside of the supergene (Fig. 8b). This clustering pattern is consistent with the supergene having originated after the divergence between *Iberoformica* and *Formica*, as documented by Brelsford et al. (2020).

*UBE4B* genes encode for large proteins ranging from 300 to 1300 amino acids in *Formica*. Conserved domains of UBE4B proteins (including the Ufd2P domain (IPR019474), which mediates polyubiquitination chain extension, and the RING/U-box domain (IPR003613), which facilitates E3 ligase interactions) are retained in most copies. Notably, specific duplicates have acquired an additional IF-rod domain (IPR039008), typically involved in cytoskeletal organization and protein scaffolding (Fig. 8c, Table S3). Protein identity within each species ranges from 10% to 100%. *Formica* species with the highest number of *UBE4B* copies tend to have more similar sequences, suggesting recent gene duplications (Figure S12). The rate of nonsynonymous to synonymous sites (dN/dS) analysis across *Formica UBE4B* gene copies indicates stronger purifying selection on the main Ufd2P and Ring/U-box domains compared to other regions of the protein. While some sites under diversifying selection are found within these conserved domains, they are more prevalent in the region between the If-rod facultative domain and the start of Ufd2P. These patterns suggest that the core functions of *UBE4B* are conserved across *Formica* species, while divergence among gene copies may allow for additional functions (Fig. 8d).

## Discussion

Supergenes and sex chromosomes, which both govern complex adaptive traits, are classically expected to degenerate once recombination is suppressed. The reduced efficacy of natural selection typically leads to haplotype contraction, accumulation of deleterious mutations, gene loss, and homozygote lethality (Bachtrog 2013; Charlesworth 2021). Yet, growing evidence shows that nonrecombining regions do not always follow this degenerative path (Berdan et al. 2021; Jeong et al. 2022; Hill et al. 2023). By comparing chromosome-scale genome assemblies of the “monogynous” and “polygynous” forms of *F. selysi*, we characterized a social supergene that, despite its old age, has taken a distinct trajectory, comprising large-scale structural expansion, bursts of TE colonization and massive gene family amplification.

Based on high quality, well-resolved assemblies for each haplotype of the social supergene of *F. selysi*, we demonstrate that the derived *P* haplotype arose from three large sequential inversions, not four as previously suggested (Brelsford et al. 2020). We inferred the relative order of inversions based on the degree of genetic differentiation and the extent of sequence expansion between haplotypes, assuming that older inversions would have accumulated more mutations and TEs compared to younger ones (Fig. 2c). According to this scenario, regions B and C belonged to the oldest inversion. A second inversion of region B and the adjacent region D subsequently resulted in the transposition of B on the *P* haplotype. The third and last large inversion comprised region A, which expanded the nonrecombining re-arrangement further, so that the supergene now spans most of chromosome 3, except for the two small recombining regions R1 and R2 that may be considered equivalent to the pseudo-autosomal regions of XY or ZW sex chromosomes (Fig. 2c).

One of the breakpoints of the most ancient inversion (with regions B and C) occurred in the LTR-rich centromeric region of chromosome 3, which disrupted the centromere integrity early in the evolution of the supergene. As a result, the centromere shifted from metacentric in the ancestral *M* haplotype to submetacentric in the *P* haplotype. Recent work suggests that these centromeric repeat-rich regions may facilitate chromosomal inversions, likely by promoting double-strand breaks and ectopic recombination (Gozashti et al. 2025). Centromere shifts due to pericentric inversions may then alter proper pairing and segregation of the ancestral and inverted homologous chromosomes during meiosis (Kirkpatrick 2010). Moreover, crossing over between ancestral and inverted haplotypes usually produces unbalanced gametes that carry deletions, insertions, and either zero or two centromeres (Thomas et al. 2008; Kirkpatrick 2010; Wellenreuther and Bernatchez 2018). Yet, despite the initial pericentric inversion, *F. selysi* queens that are heterozygous for the social supergene show no evidence of underdominance or meiotic drive (Avril et al. 2020; Blacher et al. 2023; De Gasperin et al. 2024). This confirms that some pericentric inversions escape fitness costs, for example by mechanisms like epigenetic neocentromere formation that restore proper chromosome pairing despite centromere shift (Murillo-Pineda et al. 2021; Schotanus et al. 2021) and mechanisms that suppress crossing overs during meiosis (Coyne et al. 1993; Gong et al. 2005; Kirkpatrick 2010). Regardless of the mechanisms that offset the impact of the initial pericentric inversion on fertility, the rearrangements in the social supergene of *F. selysi* are expected to significantly decrease effective recombination rates.

Population-level analyses of genetic diversity confirmed that the recombination rate was lower along the entire supergene than the rest of the genome, particularly for *MP* and *PP* individuals, as expected from the large, sequential structural rearrangement in the *P* haplotype. We found no clear difference between the inversions based on their order of appearance, showing that recombination rate does not reflect inversion age but rather the demographic and selective context in which an inversion arises and spreads. At the level of social forms, all *P*-carrying individuals present lower recombination rates in both the supergene and the rest of the genome, compared to *MM* individuals. The low recombination in the supergene is somewhat surprising for *PP* individuals, as the inverted haplotype should be able to recombine when homozygous. Overall, the genome-wide pattern of lower recombination in *P*-carrying individuals may reflect the cumulative effect of cryptic genetic load in the *P* haplotype leading to a deficit of *PP* individuals (Blacher et al. 2023), lower genetic diversity in our polygynous samples, lower effective population size in the polygynous social form (Fontcuberta et al. 2022), high rates of assortative mating by social form (Fontcuberta et al. 2021), and reduced, unidirectional gene flow between social forms (Avril et al. 2019).

Regions of low recombination tend to be rich in TEs (Dolgin and Charlesworth 2008; Duhamel et al. 2023), but it is often challenging to disentangle whether the presence of TEs facilitated genomic rearrangements (Casals et al. 2003; Zhang et al. 2009; Aasegg Araya et al. 2025), or whether TEs accumulated secondarily following such rearrangements, as a consequence of reduced recombination and relaxed selection (Fontana et al. 2020; Jay et al. 2021). In the case of the very old *Formica* supergene (Brelsford et al. 2020), breakpoint regions are now so large, complex, and TE-rich that it is difficult to identify any TEs originally involved in inversions through ectopic nonhomologous recombination. Moreover, the very high TE densities and high proportion of young TEs point toward a secondary and still ongoing accumulation of TEs in the supergene, reflecting relaxed selection for both haplotypes. TEs have accumulated, and continue to do so, more extensively in the *P* haplotype of *F. selysi*, contributing to its remarkable 27% size expansion compared to the *M* haplotype. TEs may also play a role in the cryptic load of the *P* haplotype, for example by disrupting essential genes, or altering gene expression (Lee 2022; Blacher et al. 2023; Huang and Lee 2024). The impact of TEs on load is, however, difficult to evaluate, as the reduced viability and fecundity of *P*-carrying queens and males could be due to the combined effects of many detrimental mutations, in both noncoding regulatory regions and coding regions. TEs may also contribute to the regulatory impact of the supergene by providing binding sites for transcription factors encoded within the supergene, thereby enabling genome-wide regulatory effects (Jones et al. 2025). More generally, the functional impact of TEs could be further explored by studying their expression across tissues, castes, and alternative social forms of *Formica* ants.

In ancient supergenes and sex chromosomes, prolonged suppression of recombination typically leads to gene loss in the nonrecombining, derived haplotype (Bachtrog 2013; Branco et al. 2018; Charlesworth 2021). In contrast, both haplotypes of the *F. selysi* supergene, particularly the derived, inverted *P* haplotype, show extensive gene accumulation. This is reflected in their higher gene density compared to the rest of the genome, which is largely driven by an elevated rate of gene duplication. Many duplicated genes are shared between haplotypes, suggesting they either predate the inversions or arose after the supergene rearrangement and were homogenized through rare double crossovers or gene conversion (Stevison et al. 2011; Korunes and Noor 2019; Brelsford et al. 2020). The *P* haplotype contains significantly more duplicates than the *M* haplotype, with many being haplotype-specific and belonging to shared multigenic families, suggesting that they arose through haplotype-specific duplications. Low synonymous substitution rates suggest a recent origin for most of the duplicates. The vast majority of shared and *P*-specific duplicated genes are functionally expressed, potentially reflecting sub- or neo-functionalization or increased gene dosage. In contrast, the *M* haplotype contains fewer specific duplications, and a larger fraction of them are not expressed, suggesting that purifying selection limits the retention of novel duplicates and leads to pseudogenization on this haplotype. Overall, the limited evidence for degeneration in the supergene, the viability of *PP* homozygotes (Purcell et al. 2014; Avril et al. 2019), the moderate genetic load on the *P* haplotype (Blacher et al. 2023) and the polygynous-biased expression of numerous *P*-specific duplicated genes suggest that gene gains in the *P* haplotype are adaptive and contribute to the social phenotype.

Given the large size and complexity of the supergene, pinpointing the key genetic elements and causal relationships leading to alternative social forms is challenging. In principle, any mutation impacting gene products or gene expression (i.e. substitutions, insertions, deletions, changes in gene content or in regulatory elements) could contribute to the phenotype, either individually or in combination. Moreover, key components of the supergene could regulate the expression of genes elsewhere in the genome, and even cause genome-wide effects on transcriptional activity (Jones et al. 2025). The set of previously identified trans-species candidate genes for polygyny illustrates this complexity (Brelsford et al. 2020; Purcell et al. 2021; Sigeman et al. 2025). Although most of these candidates are located within region D, this inversion is not the oldest in the supergene, indicating that key genetic elements for polygyny are not restricted to the earliest strata of suppressed recombination. At the molecular level, these genes exhibit heterogeneous signatures of divergence between social forms, including combinations of changes in coding sequences and differences in gene expression, supporting a role for both protein evolution and regulatory divergence in shaping the polygynous phenotype. Notably, most of the previously known candidates are conserved single-copy genes, showing that the trans-specific approach preferentially identified functionally constrained loci maintained across *Formica* species. This contrasts with the general trend observed at the supergene level, where gene duplication and expansion appear to be more prominent.

Among the many gene duplicates found in the supergene, the Ubiquitin Conjugation Factor E4 B *(UBE4B*) gene family stands out as a promising candidate for influencing ant social organization. Typically present as a single, highly conserved gene in insects, Hymenoptera, and other ant genera, *UBE4B* has undergone a massive expansion in the *Formica* and *Iberoformica* lineages (up to 62 copies). This expansion predates the origin of the supergene, as seven copies of *UBE4B* are found in *Iberoformica*, which lacks the supergene, and separated from *Formica* about 60 Mya (Brelsford et al. 2020). In the *Formica* lineage, *UBE4B* copies have subsequently spread across the genome, colonized the supergene, and further duplicated within each haplotype independently. In *F. selysi*, 24 and 34 *UBE4B* copies are found in the *M* and *P* haplotypes, respectively. Most of these copies remain functional, and many exhibit biased expression between social forms, suggesting functional divergence and potential roles in social phenotypes.

UBE4B is broadly recognized as a key component of the ubiquitination pathway, enabling ubiquitin-ubiquitin ligase activity and marking proteins for degradation (Hoppe 2005). In *Formica,* the core functional domains of the UBE4B proteins have been conserved by purifying selection, while other regions show signs of diversifying selection, potentially enabling novel functions. Such potential novel functions require further investigation, as the role of UBE4B in insects has not been fully characterized so far. The prevalence of functional *UBE4B* copies within the supergene suggests they play a role in the maintenance of the social phenotype. A fascinating hypothesis is that the ubiquitination pathway could contribute to alleviating some negative effects of the supergene architecture (Yu et al. 2020). For example, *UBE4B genes* may mitigate the effects of deleterious mutations that tend to accumulate on the *P* haplotype, by targeting misfolded or defective proteins for degradation.

Identifying the key genetic elements leading to polygyny, and clarifying their causal relationships, will necessitate more comparative analyses of genomes, gene expression across castes and developmental stages, and social phenotypes within and across species, followed by mechanistic and functional characterization. The *Formicidae* family provides an adequate system for such analyses, owing to its great diversity in social organization (Hölldobler and Wilson 1977; Bourke and Franks 1995) and the growing availability of genomic resources enabling detailed investigations into the genetic basis of social traits (Vizueta et al. 2025). Across and within ant species, transitions between monogynous and polygynous colony organization are associated with finely tuned adaptive variation in the morphology, physiology and behavior of both queens and workers (Bourke and Franks 1995; Ross and Keller 1995; Rosset and Chapuisat 2007; De Gasperin et al. 2024). Moreover, in at least six ant lineages, intraspecific variation in social organization, with a coexistence of monogynous and polygynous colonies, is associated with large genomic regions of low recombination, which evolved independently (Purcell et al. 2014; Kay et al. 2022; Chapuisat 2023). Convergent genetic architecture demonstrates that supergenes are major drivers of ant social organization and suggests that multiple linked genetic elements are needed to produce the multi-faceted polygynous phenotype (Kay et al. 2022; Chapuisat 2023). Identifying the functional role of each supergene element that shapes complex social phenotypes is inherently challenging. However, comparing independently evolved supergenes offers a powerful way to reveal common principles and idiosyncrasies in the genetic regulation of alternative social organizations.

Strikingly, the social supergene of *F. selysi* shares several major features with the supergene controlling colony social organization in the fire ant *Solenopsis invicta*, despite their independent origins and contrasting ages (approximately 20-40 Myr for the *Formica* supergene versus less than 1 Myr for the *Solenopsis* supergene; Purcell et al. 2014; Brelsford et al. 2020; Yan et al. 2020; Chapuisat 2023). Both supergenes exhibit remarkably similar global genetic architectures: three large inversions created two nonrecombining haplotypes, and the derived haplotypes expanded and accumulated TEs (Wang et al. 2013; Purcell et al. 2014; Stolle et al. 2019; Brelsford et al. 2020; Fontana et al. 2020; Yan et al. 2020; this study). Both supergenes harbor clusters of trans-species SNPs likely maintained by balancing selection, while extensive regions have been eroded by gene flux between haplotypes (Brelsford et al. 2020; Yan et al. 2020). In both cases, the derived haplotype is associated with polygynous social organization and distortion of transmission ratio (Keller and Ross 1998; Avril et al. 2020; Chapuisat 2023). Functionally, the “polygynous” haplotype is dominant, so heterozygotes form polygynous colonies (Chapuisat 2023), and many genes, including haplotype-specific copies, are up-regulated in polygynous individuals (Pracana et al. 2017; Cohanim et al. 2018; Arsenault et al. 2020, 2023; Jones et al. 2025). These convergent patterns indicate that similar selective pressures shaped the two independently evolved social supergenes, leading to recombination suppression across many genes, transmission ratio distortion, structural expansion, functional diversification and increased transcriptional activity.

Despite these parallels, notable differences exist between the *Formica* and *Solenopsis* social supergenes. First, the *Formica* supergene has been maintained by balancing selection through multiple speciation events (Brelsford et al. 2020; Purcell et al. 2021), whereas the *Solenopsis* supergene was assembled in one species and transferred to others by hybridization (Helleu et al. 2022; Stolle et al. 2022). Second, the *Solenopsis* supergene haplotypes differ mainly in copy number variation (CNV; Fontana et al. 2020), likely reflecting their young age, while the older *Formica* supergene haplotypes had time to accumulate more differentiated duplicates of multigene families (this study). Third, in line with their independent origins and absence of synteny (Purcell et al. 2014), the two social supergenes show pronounced functional differences. For example, the *Solenopsis* supergene is enriched for genes involved in cuticular hydrocarbon production and olfaction (Pracana et al. 2017; Cohanim et al. 2018; Fontana et al. 2020), whereas the *Formica* supergene is enriched for ubiquitin pathway genes (this study). These differences suggest that the regulation of queen number and associated traits may operate through distinct molecular mechanisms in the two lineages. However, since both social supergenes affect genome-wide expression (Jones et al. 2025; this study), it is also possible that trans-regulatory effects on genes elsewhere in the genome contribute to the similar social phenotypes. Finally, the two social supergenes differ in the extent of genetic load carried by their derived haplotypes. In *S. invicta*, homozygosity for the polygynous haplotype is lethal in queens (Gotzek and Ross 2007; Hallar et al. 2007), whereas in *F. selysi*, *PP* queens are frequent and fertile, despite evidence for cryptic lethal effects (Purcell et al. 2014; Avril et al. 2019; Blacher et al. 2023). This contrast is unexpected, as classical models predict a heavier genetic load in older supergenes (Bachtrog 2013; Llaurens et al. 2017; Charlesworth 2021; Jay et al. 2021). The moderate load in the ancient *Formica* supergene suggests that it follows an unusual evolutionary trajectory compared to many other supergenes and sex chromosomes.

The *Formica* supergene is among the oldest known autosomal supergenes, whereas most supergenes controlling complex traits, such as color morphs, mating morphs, or migratory behaviors, originated much more recently, typically within the last few million years (Wellenreuther and Bernatchez 2018; Gutiérrez-Valencia et al. 2021). The *Formica* supergene therefore represents an intermediate case between very old nonrecombining regions, like mammalian sex chromosomes (>180 Myr), and much younger supergenes or neo-sex chromosomes. Classical models predict an advanced stage of decay for ancient nonrecombining haplotypes, such as the Y chromosome (Charlesworth and Charlesworth 2000; Bachtrog 2013; Charlesworth 2016; Saunders and Muyle 2024). In contrast, the *Formica* supergene, and particularly the derived *P* haplotype, has expanded and accumulated functional gene duplicates, indicating limited degeneration despite its ancient origin. This trajectory likely results from a combination of relaxed and balancing selection. On the one hand, recombination arrest and low effective population size weakened the efficacy of selection, resulting in structural expansion, accumulation of TEs, and accumulation of gene duplicates. On the other hand, positive selection favored the evolution of novel multi-copy functional genes and purifying selection was still sufficient to prevent deleterious mutation accumulation. Occasional recombination within homozygous females, haploid selection in males, and rare double crossovers or gene conversion events (Brelsford, et al. 2020; Purcell, et al. 2021) may also limit degeneration. Together, these forces likely preserve the evolutionary potential and functional dynamism of this ancient supergene.

In summary, the large, gene-rich *Formica* supergene has persisted for millions of years under balancing selection that maintained social polymorphism. Our detailed analysis of the structure, content, and expression of each haplotype demonstrates that an ancient, recombination-reduced genomic architecture can resist extensive degeneration and even expand its functional activity over time. More broadly, these findings refine the expectation that reduced recombination inevitably leads to genetic decay, showing instead that inversion-based supergenes can remain dynamic and underpin complex phenotypes over long evolutionary timescales.

## Materials and methods

### Sample preparation and sequencing

#### Long-read sequencing

We used 30 *F. selysi* males from a single monogynous (M) colony and 20 *F. selysi* males from a single polygynous (P) colony, collected from Derborence and Finges in Valais, Switzerland, respectively (Table S4). We extracted high molecular weight DNA from the head and thorax of flash-frozen males as described in Brelsford et al. (2020) for the M males, and with Qiagen Genomic-tip 20 columns for the P males. PacBio sequencing libraries were prepared with SMRTbell template prep kits and sequenced on 26 SMRT cells of the PacBio RSII system for the M genome assembly, and on 5 SMRT cells of the PacBio Sequel system for the P genome assembly. Library preparation and sequencing were performed at the Genomic Technologies Facility (GTF) of the University of Lausanne.

#### Hi-C conformation capture

We used 19 *F. selysi* males from two monogynous colonies and 21 *F. selysi* males from two polygynous colonies, collected from Derborence in Valais, Switzerland (Table S4). To extract DNA, the head and thorax of flash-frozen males were homogenized using a CryoMill (Retsch GmbH, Haan, Germany), for 2 × 60 seconds at 25 Hz. We resuspended each finely homogenized sample in 10 mL of 1% formaldehyde solution (formaldehyde 16%, EM grade LucernaChem, diluted in MilliQ water), and transferred it into 15 mL falcon tubes. After vortexing, the samples were incubated for 20 minutes at room temperature for crosslinking (tubes were kept in horizontal position and mixed regularly by inversion). The remaining formaldehyde was sequestered by adding 100 mg of glycine to each sample tube. After vigorous vortexing, the tubes were incubated for 15 minutes at room temperature, with frequent inversion. We centrifuged samples at 1,000*×g* for 1 minute, discarded the supernatant, and rinsed the compact pellet with MilliQ water. We then resuspended the pellet in 10 mL of MilliQ water by vigorously vortexing the tube. Finally, we spun down each sample at 1,000*×g* for 1 minute and dried it on a tissue paper, after removing the supernatant. The samples were flash-frozen in liquid nitrogen and stored at −80 °C. Library preparation with Proximo Animal kit version 4.0 and Illumina sequencing (HiSeq platform, paired-end reads 2 × 150 bp) were performed by Phase Genomics (Seattle, WA, USA).

#### Short-read sequencing

We extracted DNA from 48 *F. selysi* workers collected in 48 colonies from four populations in the French and Swiss Alps (21 *MM*, 12 *MP*, 15 *PP*, Table S4) with the DNeasy Blood and Tissue kit (Qiagen, Germany), following the manufacturer's protocol for insect tissues. After removing worker's gasters, we ground the head and thorax of each worker in liquid nitrogen, using a mortar and a pestle. The resulting powder was resuspended in ATL buffer (190 μl) and Proteinase K (10 μl), and incubated overnight at 56 °C. After adding 200 μl of AL buffer and 200 μl of ethanol, we placed the samples on extraction columns and centrifuged them for 1 minute at 8,000 rpm. We discarded the flow-through and repeated the process twice, using AW1 buffer (1 minute at 8,000 rpm) and AW2 buffer (3 minutes at 14,000 rpm), respectively. We used 50 μl of AE buffer for elution. Libraries were prepared with the NEBNext Ultra II DNA kit (New England BioLabs, MA, USA) and sequenced on the Illumina HiSeq2500 platform at the Lausanne GTF, with 8 multiplexed individuals per lane. Approximately 30 million paired-end reads (2 × 100 bp) were generated by individual, leading to an average coverage of ∼22× before preprocessing.

#### RNA sequencing

We sampled workers and gynes from 21 monogynous and 38 polygynous *F. selysi* colonies in Finges, Valais, Switzerland during summers of 2016, 2017, 2018 and 2020 (Table S5). We processed some of the gynes right after emergence (virgin queen group) and other gynes after they had mated and laid their first egg in the laboratory (mated queen group). We extracted RNA from the heads and thoraxes of 20 individual workers (6 *MM*, 8 *MP*, 6 *PP*), 21 individual virgin queens (9 *MM*, 6 *MP*, 6 *PP*) and 18 individual mated queens (6 *MM*, 6 *MP*, 6 *PP*), using a Trizol-chloroform-isopropanol-based protocol. We assessed the quality of extracted RNA from each individual sample with a NanoDrop spectrophotometer (NanoDrop Technologies, Inc). Library preparation was performed at the Lausanne GTF, using the Illumina TruSeq Stranded RNA (Illumina, CA, USA) library preparation protocol. The 59 resulting libraries were sequenced on nine lanes, with 8 multiplexed individuals per lane, on the Illumina HiSeq2500 platform, yielding an average of 29 million paired-end (2 × 100 bp) reads per library (Table S5).

### Genome assemblies

We generated 86.7 Gbp (∼319× coverage) and 126.8 Gbp (∼460× coverage) of data for *F. selysi* monogynous and polygynous genome assemblies, respectively, combining PacBio long reads sequencing and Illumina short read sequencing (for Reseq and Hi-C conformation capture, Table S1). We assembled raw PacBio reads into contigs using Flye v2.7.1 (Kolmogorov et al. 2019) with the option “–genome-size 300m”. We mapped the PacBio reads against obtained contigs using pbmm2 v1.3.0 (https://github.com/PacificBiosciences/pbmm2) with “–preset SUBREAD”. We performed a first polishing step using Arrow from the GenomicConsensus v2.3.3 package GCpp v1.9.0 package (https://github.com/PacificBiosciences). We performed three additional rounds of polishing using Illumina short reads of re-sequenced individuals (Table S1) after having trimmed them using Trimmomatic v0.39 (Bolger et al. 2014) with the following parameters: ILLUMINACLIP:adapters/all.fa:2:30:12:1:true LEADING:3 TRAILING:3 MAXINFO:40:0.4 MINLEN:80. We aligned trimmed reads to the contigs using BWA mem v0.7.17 (Li 2013) and performed polishing rounds using Pilon v1.23 (Walker et al. 2014). We blasted contigs against the NBCI nt database (blastn v2.10.1+, Camacho et al. 2009) and filtered for hits with an e-value < 1e-25 (-max_target_seqs 10 -max_hsps 1). We then used BlobTools v1.0 under the taxrule “bestsumorder” (Laetsch and Blaxter 2017) to decontaminate the assemblies by removing contigs that did not correspond to metazoans taxa. Next, we mapped the raw PacBio reads against decontaminated monogynous and polygnous genome assemblies using minimap2 v2.19 (Li 2018) and filtered haplotypic duplications with Purge Haplotigs v1.1.1 (Roach et al. 2018) using the following parameters “-l 3 -m 40 -h 190” and “-l 3 -m 50 -h 190” respectively for M and P assemblies. We mapped Hi-C reads against the haploid assemblies using Juicer v1.6 (Durand et al. 2016b) with DpnII restriction site. Finally, we performed chromosome-level scaffolding with 3D-DNA v180922 (Dudchenko et al. 2017) using the parameters “-i 10,000” for the M assembly and “-i 10,000 –editor-coarse-resolution 50,000 –editor-coarse-region 250,000” for the P assembly, including a polishing step using a high-density linkage map (Brelsford et al. 2020). We visualized the resulting Hi-C contact matrix using Juicebox (Durand et al. 2016a). and assessed the completeness of genomes assemblies using BUSCO v5.1.2 (Seppey et al. 2019) against the *hymenoptera_odb10* dataset with the –long and –augustus parameters.

### Estimation of genome sizes by *k-mer* profile analyses

We trimmed the raw paired-end re-sequencing reads as described previously. We computed *k-mer* frequencies using KMC v3.0.0 (Kokot et al. 2017). We then estimated genome sizes with GenomeScope v1.0 (Vurture et al. 2017) using the parameters k-mer_length = 21 kmer_max = 1,500.

### Protein-coding gene annotation

We trimmed the raw paired-end RNA-seq reads from workers, gynes and queens of monogynous and polygynous colonies based on quality using Trimmomatic v0.39 (Bolger et al. 2014) with the maximum information approach and tolerant parameters. We discarded any reads with a sequence length of <80 bp after trimming (parameters: adapters.fa:2:30:12:1:true LEADING:3 TRAILING:3 MAXINFO:40:0.4 MINLEN:80). We mapped trimmed RNA-seq reads against the corresponding monogynous and polygynous genome assemblies using STAR v2.7.8a (Dobin et al. 2013) under the “2-pass mapping” mode with default parameters. After adding XS tag with the tagXSstrandedData.awk script following authors suggestion (Dobin et al. 2013), we produced transcriptome assemblies (for each caste and social form) using StringTie v2.1.5 (Kovaka et al. 2019). We then combined the different caste transcriptomes into a single transcriptome per social form using the Stringtie2 “merge” function. We trained and predicted protein coding genes using MAKER v2.31.8 (Holt and Yandell 2011) in a 2-iterative following Campbell et al. (2014) recommendations. For the first iteration, we predicted genes using Augustus v3.2.3 (Stanke et al. 2006) trained with BUSCO v5.1.2 results (Seppey et al. 2019). We used a combination of UniProtKB/Swiss-Prot (release 2021_02) and the BUSCO *hymenoptera_odb10* proteome as protein evidence. We used our assembled transcriptomes as transcript evidence. Following the NBIS Nextflow pipeline (Binzer-Panchal et al. 2021), we then selected complete gene models to retrain Augustus and SNAP v2013.11.29 (Korf 2004) in a second iteration. To match corresponding genes between the monogynous and polygynous form annotations, we used a reciprocal best hits (RBHs) approach. We performed blastp and blastn with Blast+ v2.12.0 (Camacho et al. 2009) for proteins and transcripts respectively, with the following parameters: “-evalue 1e-5 -max_target_seqs=1”. We considered genes with matching protein and transcript RBH in both assemblies as identical and updated their nomenclature. At this stage, 10,712 and 11,012 protein-coding genes were identified in monogynous and polygynous genome assemblies respectively (annotation v1). Among those two gene sets, 312 genes were uniquely annotated in the M assembly and 612 only in the P assembly (Figure S3). We then extracted complete gene structures (UTR-Exons-Introns) from these unique genes in Geneious Prime® 2022.2.2 and mapped them against the assembly they were absent from using the Geneious mapper in medium/fast sensitivity mode. We manually curated each mapping result, corrected annotations when necessary and finally transferred them to the respective assembly using the transfer option in Geneious. We compared multimapping due to multigenic families to find the best corresponding genes between M and P assemblies (chromosome position was also used to resolve these cases). Finally, we harmonized gene IDs between the two assemblies. The final gene set of *F. selysi* (annotation v2) consisted in a total of 11,201 genes with 10,868 and 11,107 protein-coding genes annotated in M and P assemblies, respectively, including 10,774 common genes as well as 94 and 333 genes unique to each assembly (Table 1, Figure S3). We functionally annotated predicted protein-coding genes using Blast2GO v5.5.1 (Conesa et al. 2005; Götz et al. 2008) with default parameters against the NCBI nonredundant Insecta protein database (v2021-08).

### Centromere location prediction

To predict centromere location in *F. selysi* chromosomes, we combined several approaches. We first used blastn (blast+ v2.14.1, e-value≤1e-5) to map five satellite DNA sequences identified in *F. selysi* (Lorite et al. 2004, AJ508850.1, AJ508849.1, AJ508848.1, AJ508847.1, AJ508846.1) to the M and P genome assemblies. As this previous study may not have identified all possible satellite DNAs of *F. selysi*, we then predicted centromeres location using CentIER (Xu et al. 2024). CentIER was originally designed for plant genomes but successfully predicted centromeres in the fire ant *Solenopsis invicta* (Figure S13a). Predicted centromeres overlapped with the 10,000 identified satellite DNAs in *S. invicta* (*CenSol*, Huang et al. 2016). Predicted centromere sizes appeared shorter than previously characterized, which indicates a slight underestimation by CentIER (Figure S13a). In *F. selysi*, the low number of identified satellite DNAs generated few mappings on the genome which explains a weaker overlap between these and centromere prediction by CentIER (Figures S13b and c). Finally, to explore and visualize predicted centromeres, we created dotplots for each chromosome aligned to itself. If a genomic region is highly repetitive (such as centromeres), self-alignments are numerous, and this region appears denser in the dotplot. We used nucmer with –maxmatch, -l 50, -c 100 to align and plotted all alignments >100 bp in R. We also calculated self-alignment density across 100 kb overlapping windows (step = 25 kb) and defined high-density regions as the longest stretches of consecutive windows with more than 20% self-alignment (Figure S14). For *F. selysi* chromosomes 3, we defined centromere confidence intervals encompassing CentIER predictions, satellite DNA matches and regions with highest self-alignment densities. We finally checked if the centromere confidence intervals corresponded to regions with low gene density and reversely high TE and tandem repeat (TR) densities.

### TE analysis

To investigate TE distribution in *F. selysi*, we generated a comprehensive TE annotation for each assembly. We modeled TE de novo with RepeatModeler v2.0.2a (Smit et al. 2008) using the option -LTRStruct. We identified and filtered out potential transposon ORFs and protein-coding genes using TransposonPSI v08222010 (http://transposonpsi.sourceforge.net/) and ProtExcluder v1.2 (Campbell et al. 2014). We combined the de novo repeats with known TEs found in insects from the databases Dfam v3.1 (Storer et al. 2021) and RepBase (Bao et al. 2015). We annotated TEs across each assembly with RepeatMasker v4.1.0 (Tarailo-Graovac and Chen 2009) using the species-specific merged TE library. We then postprocessed RepeatMasker outputs using the “One code to find them all” script (Bailly-Bechet et al. 2014) with default parameters to extract TE families and genomic positions. We determined TE count and span (in bp) within chromosomes and fixed windows along genome assemblies (10 and 50 kb) using BEDTools coverage (Quinlan 2014) for all TEs grouped together and separately for DNA transposons, long interspersed nuclear elements retrotransposons (LINE), LTR, rolling-circle transposons (RC), short interspersed nuclear elements retrotransposons (SINE) and unclassified TEs. To investigate TE age distribution, we ran separately RepeatMasker for each region of the supergene and the rest of the genome and parse the output with parseRM_GetLandscape.pl (https://github.com/4ureliek/Parsing-RepeatMasker-Outputs). We used the CpG-adjusted Kimura distance of each TE insertion from its consensus sequence as a proxy for TE age and chose a distance inferior at 10% as threshold for recent TEs.

### Gene orthology analysis

We defined orthologous gene groups between *F. selysi* and seven other Formicidae species: *Harpegnathos saltator* (Bonasio et al. 2010; Shields et al. 2018), *Ooceraea biroi* (McKenzie and Kronauer 2018), *Acromyrmex echinatior* (Nygaard et al. 2011), *Solenopsis invicta* (Yan et al. 2020), *Pogonomyrmex barbatus* (Smith et al. 2011), *Camponotus floridanus* (Bonasio et al. 2010; Shields et al. 2018), and *Formica exsecta* (Dhaygude et al. 2019). *A. mellifera* (Wallberg et al. 2019) and *D. melanogaster* (Hoskins et al. 2015) were added as outgroups. Proteomes for these species were extracted from NCBI RefSeq (January 2021). We first filtered the longest isoform for each protein-coding gene and then ran OrthoFinder v2.5.4 (Emms and Kelly 2015; Emms and Kelly 2019) using defaults parameters. We selected 4,462 single-copy orthologue groups conserved across the 10 species, extracted the protein sequences, aligned them with MAFFT v7 (Katoh and Standley 2013), and concatenated the alignments into one. We built species phylogeny from the alignment using RAxML v8.2.12 (Stamatakis 2006; Stamatakis et al. 2008) with the following parameters: -m PROTGAMMAAUTO -p 12345 -x 12345 -# 1000. We determined categories of orthology for genes based on their presence-absence in different orthologue groups across species. We compared orthologous gene categories between *F. selysi* whole gene set and genes within the M and P haplotypes of the supergene by Chi-square test for each category followed by Bonferroni correction and computed average *P* values for global distribution comparisons. Finally, category enrichment for *M* and *P* haplotype was calculated by resampling 10,000 times the distribution of *F. selysi* orthologous gene categories for the number of genes present in each haplotype (588 and 764 genes for *M* and *P,* respectively). We obtained *P* values by dividing the number of iterations showing simulated values greater than the observed number of genes in each category by 10,000.

### Gene density and duplication analyses

To define single- and multiple-copy genes, we extracted *F. selysi* gene counts from orthologue groups previously identified by Orthofinder. Genes were classified as single-copy if present as a single instance in both M and P assemblies, whereas those with two or more copies in one or both assemblies were categorized as multiple-copy genes. We calculated overall gene density (i.e. total gene number) and duplication rate (i.e. number of multiple-copy genes) in 50-kb fixed windows across the M and P genome assemblies and different supergene haplotype regions using BEDTools coverage (Quinlan 2014). To assess enrichment or depletion of single- and multiple-copy genes within the supergene haplotypes, we performed 10,000 resampling iterations of the number of genes present in each haplotype within the genome-wide distribution of *F. selysi* single- and multiple-copy genes. We computed *P* values by dividing the number of iterations showing simulated values greater or lower than observed number of genes in each category by 10,000. For complex orthogroups containing more than two genes, we identified duplicated gene pairs via all-against-all blastn (BLAST+ v2.14.1) within each orthogroup. Paralogous pairs were selected based on the highest bit-scores and lowest e-values using a custom Python script. Coding sequences of these duplicated gene pairs were extracted and aligned with MACSE (Ranwez et al. 2011). From these codon alignments, we estimated pairwise synonymous substitution rates (dS) using the YNOO model in PAML v4.8 (Yang and Nielsen 2000; Yang 2007). To ensure reliability, we filtered out gene pairs with saturated dS values (>2) to exclude potential misalignments and erroneous estimates.

### Gene expression analysis

To compare gene expression across castes and social forms (in adult females), we trimmed RNA-seq reads based on quality using Trimmomatic v0.39 (Bolger et al. 2014) and pseudo-aligned them on *F. selysi* transcriptome with Kallisto v0.48 (Bray et al. 2016). The reference transcriptome contained transcripts from the 10,774 common genes (M transcript versions) and the 94 and 333 transcripts unique to the M and P assemblies, respectively. We kept all the different alternative transcripts for each gene, resulting in a reference with 12,397 transcripts. After pseudo-alignment, we collapsed estimated counts from Kallisto for each gene, computed count per million (CPM) and kept the genes with CPM>1 in at least 6 samples (minimal number of replicates per condition). We then checked samples correlation by MDS clustering and normalized estimated counts with trimmed mean of M values (TMM) method (Robinson and Oshlack 2010). To call DEGs between the castes and social forms, we estimated variance of log-transformed normalized counts and computed weights with voom function from limma package (Law et al. 2014; Ritchie et al. 2015, Figure S15). We fitted linear models for each gene using lmFit and computed statistics for each contrast (caste × genotype) with eBayes. Benjamini-Hochberg false discovery rate was finally used to adjust *P* values. We retained as DEGs genes with a log2(fold-change) ≥ 2 and an adjusted *P* value of ≤0.05. To test if the supergene is enriched in DEGs, we calculated the ratio of observed DEGs over expected DEGs based on supergene and genome lengths, respectively, and computed chi-square tests.

### Previously identified candidate gene analysis

For the 13 polygyny candidate genes previously identified through comparative analyses across *Formica* species (Brelsford et al. 2020; Purcell et al. 2021; and Sigeman et al. 2025), we extracted genome location, orthology patterns, expression patterns and gene evolution metrics (dN/dS and pN/pS) from the genome-wide analyses conducted in *F. selysi*. To assess differences between *M* and *P* alleles, we aligned protein sequences with ClustalW v1.2.3 (Sievers and Higgins 2018) and evaluated the impact of both single amino-acid substitutions and larger exon deletions on the protein structure and function.

### *UBE4B* gene analysis

We first extracted protein sequences encoded by the Ubiquitin conjugation factor E4 B (*UBE4B*) genes from the orthogroups generated by an Orthofinder analysis across *F. selysi* and nine other species genomes. We then identified the orthogroup corresponding to the UBE4B family within the genome data generated by the Global Ant Genomics Alliance (GAGA; Vizueta et al. 2025) and retrieved UBE4B proteins from five additional *Formica* species (*Formica japonica, Formica cf. japonica, Formica sanguinea, Formica fusca, Formica cinerea*) and from *Iberoformica subrufa*  Table S3). We aligned a total of 252 UBE4B protein sequences with ClustalW v1.2.3 (Sievers and Higgins 2018) and generated a maximum-likelihood (ML) phylogenetic tree using RAxML-NG v.1.0.0 (doi:10.5281/zenodo.593079) under the ML tree search + bootstrapping mode using the following parameters: –all –model LG + G4 –seed 12345 –bs-trees 1000. We ran InterProScan (Quevillon et al. 2005) to identify protein domains within the 252 UBE4B proteins. We visualized and annotated the tree using iTOL v6 (Letunic and Bork 2024). To assess evidence for both episodic and pervasive selection in UBE4B genes within *Formica*, we first retrieved the coding sequences (CDS) of all gene copies from each species. These sequences were aligned in-frame using TransAlign (Geneious Prime® 2022.2.2) to preserve codon structure. The resulting codon alignment was analyzed with the MEME method (Mixed Effects Model of Evolution, Murrell et al. 2012) to detect sites under episodic diversifying selection, and the FUBAR method (Fast Unconstrained Bayesian AppRoximation, Murrell et al. 2013) to identify sites under pervasive diversifying or purifying selection. Both analyses were performed on the DATAMONKEY online server (www.datamonkey.org, Weaver et al. 2018). We calculated the mean dN/dS ratio from FUBAR estimates in nonoverlapping 15-codon windows, and integrated MEME and FUBAR results to identify codons under diversifying selection. We used significance thresholds of *P* < 0.05 and *P* < 0.01 for MEME, while FUBAR results were considered significant for posterior probabilities >0.95 or >0.99. Gene Ontology (GO) terms enrichment analyses were conducted with GO.db (Carlson 2024) and topGO (Alexa and Rahnenführer 2024) packages using R Statistical Software (v4.1.2; R Core Team 2021).

### Variant calling and population genetic tests with resequencing data

We quality checked the reads generated from the resequencing of 48 *F. selysi* individuals from four populations, using FastQC 0.11. We then quality-trimmed reads using Trimmomatic v0.39 (Bolger et al. 2014) adapters.fa:2:30:12:1:true LEADING:3 TRAILING:3 MAXINFO:40:0.4 MINLEN:80). Fragments shorter than 80 bp were discarded. We then aligned the paired reads to the M and P genome assemblies using the bwa-mem algorithm (BWA v.0.7.17, (Li 2013)). We marked and removed sequencing duplicates using Picard tools v2.21.8, following which, we recalibrated base calls using the BaseRecalibrator in the Genome Analysis Toolkit (GATK) pipeline (GATK v4.2.0.0; McKenna et al. 2010). We then called variants using the HaplotypeCaller in the GATK and combined all the samples into a single vcf file. We split the resulting variants into INDELs and SNPs and filtered the SNPs using the VariantFiltration in the GATK, with the following parameters: QD < 2.0, SOR > 3.0, FS > 60.0, MQ < 40.0, MQRankSum < −12.5, ReadPosRankSum < −8.0. We calculated Weir and Cockerham's *F*_st_ for SNPs on chromosome 3 using vcftools 0.1.15 (Danecek et al. 2011). We compared the median *F*_st_ values for each region on chromosome 3 with each other using Wilcoxon rank sum test with Bonferroni correction for multiple testing. We also compared the median *F*_st_ values for each region on chromosome 3 with randomly sampled sites from across the chromosome using the Kruskal-Wallis rank sum test.

To estimate recombination rate across chromosomes in the M and P assemblies, we phased the SNPs on each chromosome using SHAPEIT4 (Delaneau et al. 2019) and calculated recombination rate (rho) for groups of individuals of each social supergene genotype using the FastEPRR package in R (Gao et al. 2016). We used sliding windows of 100 kb with 20 kb steps and rho values of trainingSet1. We compared recombination rates for each region of chromosome 3 with each other and with the rest of the genome using a pairwise Dunn's post hoc test with Bonferroni correction for multiple comparisons.

### Calculating direction of selection for supergene haplotypes

To estimate direction of selection (DoS) between the *M* and *P* supergene haplotypes compared to the rest of the genome, we calculated pN/pS and dN/dS for 10,620 single-copy coding genes and multi-copy genes with equivalent homologs in both M and P assemblies. We used *Polyergus mexicanus* and *Polyergus vinosus* as monomorphic monogynous outgroups to the *Formica* clade. We downloaded sequences for these species from the SRA database (accession numbers SRR15845476 and SRR15845487) and processed them using the pipeline described above for the resequencing data. We then converted the aligned sequences to FASTA format, extracted and concatenated coding regions for single-copy genes in all *F. selysi* and *Polyergus* samples using samtools 1.15.1 (Li et al. 2009), gffread 0.12.7 (Pertea and Pertea 2020) and Seqkit 0.13.2 (Shen et al. 2024). We then calculated dN/dS between the two species and pN/pS within *F. selysi* samples using SNPGenie (Nelson et al. 2015). For 11 genes in the M assembly that had truncated sequences (not in multiples of three bp), SNPGenie was unable to calculate dN/dS and pN/pS ratios. We dropped these genes from our analysis. We calculated the two ratios for each gene and pooled them into 500 kb windows for each chromosome in the genome. We calculated DoS using the following formula *DoS* = *dN/(dN*  *+*  *dS)-pN/(pN*  *+*  *pS)*. We used the Wilcoxon rank sum test with Bonferroni correction for multiple testing to compare the median values for dN/dS, pN/pS and DoS across each region on chromosome 3 with each other and with the rest of the genome.

## Supplementary Material

msag127_Supplementary_Data

## Data Availability

Raw PacBio and Hi-C sequencing data used for genome assembly, transcriptome sequencing data used for gene expression analyses, and whole-genome resequencing data used for molecular evolution and recombination rate analyses have been deposited in the NCBI Sequence Read Archive under BioProject PRJNA1347246. Genome assemblies and annotations of the monogynous and polygynous forms of *F. selysi* are available under accessions JBTLQM000000000 and JBTLQL000000000. The analyses were performed with publicly available tools and software, all of which are cited in the Materials and Methods section. Analysis pipelines and commands are available on Zenodo (https://doi.org/10.5281/zenodo.19128538).
