# Supplementary material for: Ancient but dynamic: structural expansion, massive gene duplication, and transposable element colonization in a supergene controlling ant social organization
Source: Mol Biol Evol. 2026 May 27;43(6):msag127. doi: 10.1093/molbev/msag127 (PMC13251583; doi:10.1093/molbev/msag127)
Supplement: msag127_Supplementary_Data [file msag127_supplementary_data.pdf]

Supplementary Tables and Figures

Ancient but dynamic: structural expansion, massive gene duplication, and transposable element colonization in a supergene controlling ant social organization

Hélène Boulain[1]*, Riddhi Deshmukh[1]*, Amaury Avril[1], Pierre Blacher[1], Massimo Bourquin[1], Sze Huei Yek[1,2], Sacha Zahnd[1], Patrick Tran Van[1], Michel Chapuisat[1]

[1]Department of Ecology and Evolution, University of Lausanne, 1015 Lausanne, Switzerland
[2]Present address: Institute for Tropical Biology and Conservation, Universiti Malaysia Sabah, 88400 Kota Kinabalu, Malaysia
*These authors contributed equally

Table S1. Data statistics for the reference genome assemblies

Sample ID	Genotype	Data type<br>Step	Yield [Gbp]	Coverage [X]
DE90_pool_M	M	PacBio RS II<br>Contig assembly	30.4	112.1
De115W1		Illumina HiSeq 2500 2x101 bp<br>Polishing+ k-mer size estimation	5.0	18.5
De67W1	MM	Illumina HiSeq 2500 2x101 bp<br>Polishing+ k-mer size estimation	5.4	19.8
De84W1		Illumina HiSeq 2500 2x101 bp<br>Polishing+ k-mer size estimation	7.3	26.8
FsiM_HiC	M	Illumina HiSeq ref 2x150 bp<br>Hi-C Scaffolding	38.6	142.3
FI43_pool_P	P	PacBio Sequel<br>Contig assembly	35.7	128.8
703W4		Illumina HiSeq 2500 2x101 bp<br>Polishing + k-mer size estimation	5.3	19.0
722W1	PP	Illumina HiSeq 2500 2x101 bp<br>Polishing+ k-mer size estimation	5.5	20.0
733W1		Illumina HiSeq 2500 2x101 bp<br>Polishing+ k-mer size estimation	5.2	18.9
FsiP_HiC	P	Illumina HiSeq ref 2x150 bp<br>Hi-C Scaffolding	75.1	271.3

Read coverage for the reference genome assemblies was estimated using the average haploid genome size predicted by the k-mer analyses. bp: basepairs, G: Giga.

Table S2. *UBE4B* genes in *Formica selysi*

Gene ID number	Gene ID M assembly	Gene ID P assembly	Location M assembly	Location P assembly	Annotation status	Blas2GO annotation	Expression bias	IF domain*	UFd2P domain*	U-box domain*
ID_000129	FSIM_S1_000129	FSIP_S1_000129	scaf	scaf	shared	Ubiquitin_conjugation_factor_E4_B	Mono_biased	no	Yes	Yes
ID_001064	FSIM_S2_001064	FSIP_S2_001064	scaf	scaf	shared	ubiquitin_conjugation_factor_E4_B-like	ns	no	Yes	no
ID_001150	FSIM_S2_001150	FSIP_S2_001150	scaf	scaf	shared	Ubiquitin_conjugation_factor_E4_B	ns	no	Yes	Yes
ID_001154	FSIM_S2_001154	FSIP_S2_001154	scaf	scaf	shared	Ubiquitin_conjugation_factor_E4_B	ns	no	Yes	Yes
ID_001160	FSIM_S2_001160	FSIP_S2_001160	scaf	scaf	shared	ubiquitin_conjugation_factor_E4_B	ns	no	Yes	Yes
ID_001434	FSIM_S3A_001434	NA	supergene	NA	M-specific	ubiquitin_conjugation_factor_E4_B-like	Mono_biased	no	Yes	Yes
ID_001435	FSIM_S3A_001435	NA	supergene	NA	M-specific	Ubiquitin_conjugation_factor_E4_B	Mono_biased	no	Yes	Yes
ID_001452	FSIM_S3A_001452	FSIP_S3A_001452	supergene	supergene	shared	ubiquitin_conjugation_factor_E4_B-like	ns	no	Yes	Yes
ID_001453	FSIM_S3A_001453	FSIP_S3A_001453	supergene	supergene	shared	Ubiquitin_conjugation_factor_E4_B	ns	no	Yes	Yes
ID_001458	FSIM_S3A_001458	FSIP_S3A_001458	supergene	supergene	shared	ubiquitin_conjugation_factor_E4_B-like	ns	no	Yes	Yes
ID_001461	FSIM_S3A_001461	FSIP_S3A_001461	supergene	supergene	shared	Ubiquitin_conjugation_factor_E4_B	ns	no	Yes	Yes
ID_001477	FSIM_S3A_001477	FSIP_S3A_001477	supergene	supergene	shared	Ubiquitin_conjugation_factor_E4_B	ns	no	Yes	Yes
ID_001482	FSIM_S3A_001482	NA	supergene	NA	M-specific	Ubiquitin_conjugation_factor_E4_B	ns	no	Yes	Yes
ID_001483	FSIM_S3A_001483	FSIP_S3A_001483	supergene	supergene	shared	Ubiquitin_conjugation_factor_E4_B	Mono_biased	no	Yes	Yes
ID_001527	FSIM_S3A_001527	NA	supergene	NA	M-specific	ubiquitin_conjugation_factor_E4_B-like	Mono_biased	no	Yes	no
ID_001673	FSIM_S3A_001673	NA	supergene	NA	M-specific	Ubiquitin_conjugation_factor_E4_B	Mono_biased	Yes	Yes	Yes
ID_001675	FSIM_S3A_001675	NA	supergene	NA	M-specific	Ubiquitin_conjugation_factor_E4_B	Mono_biased	Yes	Yes	Yes
ID_001815	FSIM_S3C_001815	FSIP_S3C_001815	supergene	supergene	shared	ubiquitin_conjugation_factor_E4_B-like	Mono_biased	Yes	Yes	Yes
ID_001883	FSIM_S3D_001883	FSIP_S3C_001883	supergene	supergene	shared	Ubiquitin_conjugation_factor_E4_B	Mono_biased	Yes	Yes	Yes
ID_001898	FSIM_S3D_001898	FSIP_S3D_001898	supergene	supergene	shared	Ubiquitin_conjugation_factor_E4_B	ns	no	Yes	Yes
ID_001901	FSIM_S3D_001901	FSIP_S3D_001901	supergene	supergene	shared	ubiquitin_conjugation_factor_E4_B-like	ns	no	Yes	Yes
ID_001917	FSIM_S3D_001917	FSIP_S3D_001917	supergene	supergene	shared	ubiquitin_conjugation_factor_E4_B-like	ns	Yes	Yes	Yes
ID_001938	FSIM_S3D_001938	FSIP_S3D_001938	supergene	supergene	shared	ubiquitin_conjugation_factor_E4_B_isoform_X2	Mono_biased	no	Yes	Yes
ID_001950	FSIM_S3D_001950	FSIP_S3D_001950	supergene	supergene	shared	ubiquitin_conjugation_factor_E4_B_isoform_X2	ns	no	Yes	Yes
ID_001972	FSIM_S3D_001972	NA	supergene	NA	M-specific	ubiquitin_conjugation_factor_E4_B-like	Mono_biased	no	Yes	Yes
ID_001976	FSIM_S3D_001976	FSIP_S3D_001976	supergene	supergene	shared	Ubiquitin_conjugation_factor_E4_B	Mono_biased	Yes	Yes	Yes
ID_001991	FSIM_S3D_001991	FSIP_C1204_001991	supergene	contigs	shared	Ubiquitin_conjugation_factor_E4_B	Mono_biased	Yes	Yes	Yes
ID_002007	FSIM_S3D_002007	FSIP_S3D_002007	supergene	supergene	shared	ubiquitin_conjugation_factor_E4_B	Mono_biased	no	Yes	Yes
ID_002017	FSIM_S3_002017	NA	supergene	NA	M-specific	ubiquitin_conjugation_factor_E4_B-like	ns	Yes	Yes	no
ID_002103	FSIM_S4_002103	FSIP_S4_002103	scaf	scaf	shared	Ubiquitin_conjugation_factor_E4_B	ns	no	Yes	Yes
ID_002104	FSIM_S4_002104	FSIP_S4_002104	scaf	scaf	shared	Ubiquitin_conjugation_factor_E4_B	ns	no	Yes	Yes
ID_003996	FSIM_S8_003996	FSIP_S8_003996	scaf	scaf	shared	Ubiquitin_conjugation_factor_E4_B	ns	no	Yes	Yes
ID_005390	FSIM_S11_005390	FSIP_S11_005390	scaf	scaf	shared	Ubiquitin_conjugation_factor_E4_B	ns	no	Yes	Yes
ID_006101	FSIM_S13_006101	FSIP_S13_006101	scaf	scaf	shared	Ubiquitin_conjugation_factor_E4_B	ns	no	Yes	Yes
ID_006137	FSIM_S13_006137	FSIP_S13_006137	scaf	scaf	shared	Ubiquitin_conjugation_factor_E4_B	ns	no	Yes	Yes
ID_006148	FSIM_S13_006148	FSIP_S13_006148	scaf	scaf	shared	Ubiquitin_conjugation_factor_E4_B	ns	no	Yes	Yes
ID_006726	FSIM_S14_006726	FSIP_S14_006726	scaf	scaf	shared	Ubiquitin_conjugation_factor_E4_B	ns	no	Yes	Yes
ID_006752	FSIM_S14_006752	FSIP_S14_006752	scaf	scaf	shared	Ubiquitin_conjugation_factor_E4_B	ns	no	Yes	Yes
ID_010394	FSIM_S26_010394	FSIP_S26_010394	scaf	scaf	shared	Ubiquitin_conjugation_factor_E4_B	Mono_biased	no	Yes	Yes
ID_010927	NA	FSIP_C1089_010927	NA	contigs	P-specific	Ubiquitin_conjugation_factor_E4_B	Poly_biased	Yes	Yes	Yes
ID_010936	NA	FSIP_C1709_010936	NA	contigs	P-specific	Ubiquitin_conjugation_factor_E4_B	Poly_biased	no	Yes	Yes
ID_010949	NA	FSIP_C3_010949	NA	contigs	P-specific	Ubiquitin_conjugation_factor_E4_B	Poly_biased	no	Yes	Yes

ID_010962	NA	FSIP_C957_010962	NA	contigs	P-specific	Ubiquitin_conjugation_factor_E4_B	Poly_biased	Yes	Yes	Yes
ID_010963	NA	FSIP_C957_010963	NA	contigs	P-specific	Ubiquitin_conjugation_factor_E4_B	Poly_biased	no	Yes	Yes
ID_011087	NA	FSIP_S3D_011087	NA	supergene	P-specific	ubiquitin_conjugation_factor_E4_B_isoform_X1	Poly_biased	no	Yes	Yes
ID_011121	NA	FSIP_S3_011121	NA	supergene	P-specific	Ubiquitin_conjugation_factor_E4_B	Poly_biased	no	Yes	Yes
ID_011122	NA	FSIP_S3_011122	NA	supergene	P-specific	Ubiquitin_conjugation_factor_E4_B	ns	Yes	Yes	Yes
ID_011125	NA	FSIP_S3A_011125	NA	supergene	P-specific	Ubiquitin_conjugation_factor_E4_B	Poly_biased	no	Yes	Yes
ID_011126	NA	FSIP_S3A_011126	NA	supergene	P-specific	ubiquitin_conjugation_factor_e4_b	Poly_biased	no	Yes	no
ID_011127	NA	FSIP_S3A_011127	NA	supergene	P-specific	Ubiquitin_conjugation_factor_E4_B	Poly_biased	no	Yes	Yes
ID_011128	NA	FSIP_S3A_011128	NA	supergene	P-specific	Ubiquitin_conjugation_factor_E4_B	Poly_biased	Yes	Yes	Yes
ID_011129	NA	FSIP_S3A_011129	NA	supergene	P-specific	Ubiquitin_conjugation_factor_E4_B	Poly_biased	no	Yes	Yes
ID_011131	NA	FSIP_S3A_011131	NA	supergene	P-specific	Ubiquitin_conjugation_factor_E4_B	Poly_biased	no	Yes	Yes
ID_011132	NA	FSIP_S3A_011132	NA	supergene	P-specific	Ubiquitin_conjugation_factor_E4_B	Poly_biased	no	Yes	Yes
ID_011133	NA	FSIP_S3A_011133	NA	supergene	P-specific	Ubiquitin_conjugation_factor_E4_B	Poly_biased	no	Yes	Yes
ID_011163	NA	FSIP_S3C_011163	NA	supergene	P-specific	Ubiquitin_conjugation_factor_E4_B	Poly_biased	Yes	Yes	Yes
ID_011164	NA	FSIP_S3C_011164	NA	supergene	P-specific	Ubiquitin_conjugation_factor_E4_B	Poly_biased	no	Yes	Yes
ID_011165	NA	FSIP_S3C_011165	NA	supergene	P-specific	Ubiquitin_conjugation_factor_E4_B	Poly_biased	no	Yes	Yes
ID_011166	NA	FSIP_S3C_011166	NA	supergene	P-specific	Ubiquitin_conjugation_factor_E4_B	Poly_biased	Yes	Yes	Yes
ID_011167	NA	FSIP_S3C_011167	NA	supergene	P-specific	Ubiquitin_conjugation_factor_E4_B	Poly_biased	no	Yes	Yes
ID_011188	NA	FSIP_S3D_011188	NA	supergene	P-specific	Ubiquitin_conjugation_factor_E4_B	Poly_biased	no	Yes	Yes
ID_011189	NA	FSIP_S3D_011189	NA	supergene	P-specific	Ubiquitin_conjugation_factor_E4_B	Poly_biased	Yes	Yes	Yes

*Protein domains identified by InterProScan. Mono_biased = Up-regulated expression in monogynous, Poly_biased = Up-regulated expression in polygynous, ns= not significant.

Table S3. *UBE4B* genes in *Formica* and outgroup species

ID	Species	IF domain*	UFd2P domain*	U-box domain*
XP_011065650.1	*Acromyrmex echinatior*	no	Yes	Yes
XP_006566703.1	*Apis mellifera*	no	Yes	Yes
XP_011265548.1	*Camponotus floridanus*	no	Yes	Yes
NP_609597.1	*Drosophila melanogaster*	no	Yes	Yes
Ffus_g00510_i1	*Formica cf. japonica*	no	Yes	Yes
Ffus_g02689_i1	*Formica cf. japonica*	no	Yes	Yes
Ffus_g03289_i1	*Formica cf. japonica*	no	Yes	Yes
Ffus_g03290_i1	*Formica cf. japonica*	no	Yes	Yes
Ffus_g03369_i1	*Formica cf. japonica*	no	Yes	Yes
Ffus_g04184_i1	*Formica cf. japonica*	no	Yes	Yes
Ffus_g06159_i1	*Formica cf. japonica*	Yes	Yes	Yes
Ffus_g07411_i1	*Formica cf. japonica*	Yes	no	no
Ffus_g07461_i1	*Formica cf. japonica*	Yes	Yes	Yes
Ffus_g07463_i1	*Formica cf. japonica*	Yes	Yes	no
Ffus_g08080_i1	*Formica cf. japonica*	no	Yes	Yes
Ffus_g09494_i2	*Formica cf. japonica*	no	Yes	no
Ffus_g09499_i1	*Formica cf. japonica*	no	Yes	Yes
Ffus_g09564_i2	*Formica cf. japonica*	Yes	Yes	Yes
Ffus_g10464_i1	*Formica cf. japonica*	Yes	Yes	Yes
Ffus_g10782_i1	*Formica cf. japonica*	no	Yes	Yes
Ffus_g10788_i2	*Formica cf. japonica*	no	Yes	Yes
Ffus_g10789_i1	*Formica cf. japonica*	no	Yes	Yes
Ffus_g10798_i1	*Formica cf. japonica*	Yes	Yes	Yes
Ffus_g10829_i1	*Formica cf. japonica*	no	Yes	Yes
Ffus_g10940_i1	*Formica cf. japonica*	no	Yes	no
Ffus_g11016_i1	*Formica cf. japonica*	no	Yes	Yes
Ffus_g11339_i1	*Formica cf. japonica*	no	Yes	Yes
Ffus_g11687_i1	*Formica cf. japonica*	Yes	Yes	Yes
Ffus_g11847_i1	*Formica cf. japonica*	no	Yes	Yes
Ffus_g12505_i1	*Formica cf. japonica*	no	Yes	Yes
Ffus_g12528_i1	*Formica cf. japonica*	no	Yes	Yes
Ffus_g12548_i1	*Formica cf. japonica*	no	no	no
Ffus_g13074_i1	*Formica cf. japonica*	no	Yes	Yes
Ffus_g13082_i1	*Formica cf. japonica*	no	Yes	Yes
Ffus_g13083_i1	*Formica cf. japonica*	no	Yes	Yes
Ffus_g13101_i1	*Formica cf. japonica*	Yes	Yes	Yes
Ffus_g13103_i1	*Formica cf. japonica*	no	Yes	Yes
Ffus_g13130_i4	*Formica cf. japonica*	Yes	Yes	Yes
Ffus_g13935_i1	*Formica cf. japonica*	no	Yes	Yes
Ffus_g14336_i1	*Formica cf. japonica*	no	Yes	Yes
Ffus_g14339_i3	*Formica cf. japonica*	Yes	Yes	Yes
Ffus_g14340_i1	*Formica cf. japonica*	Yes	Yes	Yes
Ffus_g14373_i1	*Formica cf. japonica*	Yes	Yes	no
Ffus_g14394_i1	*Formica cf. japonica*	Yes	Yes	Yes
Ffus_g14400_i2	*Formica cf. japonica*	Yes	Yes	Yes
Ffus_g16243_i1	*Formica cf. japonica*	no	Yes	Yes
Ffus_g16291_i1	*Formica cf. japonica*	no	Yes	no
Ffus_g16371_i2	*Formica cf. japonica*	Yes	Yes	Yes
Ffus_g16396_i2	*Formica cf. japonica*	Yes	Yes	Yes
Ffus_g16798_i1	*Formica cf. japonica*	no	Yes	Yes
Ffus_g17178_i1	*Formica cf. japonica*	no	Yes	Yes
Ffus_g17323_i1	*Formica cf. japonica*	no	Yes	Yes
Ffus_g17464_i1	*Formica cf. japonica*	no	Yes	Yes
Ffus_g17465_i1	*Formica cf. japonica*	no	Yes	Yes

Fcin_g00053_i1	*Formica cinerea*	no	Yes	no
Fcin_g01195_i1	*Formica cinerea*	no	Yes	Yes
Fcin_g02408_i1	*Formica cinerea*	no	Yes	Yes
Fcin_g02409_i1	*Formica cinerea*	Yes	Yes	Yes
Fcin_g02410_i1	*Formica cinerea*	no	Yes	Yes
Fcin_g02496_i1	*Formica cinerea*	no	Yes	Yes
Fcin_g02497_i1	*Formica cinerea*	no	Yes	Yes
Fcin_g02515_i1	*Formica cinerea*	no	Yes	Yes
Fcin_g02574_i1	*Formica cinerea*	Yes	Yes	Yes
Fcin_g02590_i1	*Formica cinerea*	Yes	no	no
Fcin_g02609_i1	*Formica cinerea*	no	Yes	no
Fcin_g04071_i2	*Formica cinerea*	no	Yes	Yes
Fcin_g04074_i1	*Formica cinerea*	no	Yes	Yes
Fcin_g04466_i1	*Formica cinerea*	no	Yes	Yes
Fcin_g10923_i1	*Formica cinerea*	no	Yes	Yes
Fcin_g10967_i1	*Formica cinerea*	no	Yes	Yes
Fcin_g10983_i1	*Formica cinerea*	no	Yes	Yes
Fcin_g10992_i1	*Formica cinerea*	no	Yes	Yes
Fcin_g11013_i1	*Formica cinerea*	no	Yes	Yes
Fcin_g14265_i1	*Formica cinerea*	no	Yes	Yes
Fcin_g14330_i1	*Formica cinerea*	no	Yes	Yes
Fcin_g15675_i1	*Formica cinerea*	no	Yes	Yes
Fcin_g15681_i1	*Formica cinerea*	no	Yes	Yes
Fcin_g15786_i1	*Formica cinerea*	no	Yes	Yes
Fcin_g15795_i1	*Formica cinerea*	no	Yes	Yes
Fcin_g16398_i1	*Formica cinerea*	no	Yes	Yes
XP_029659303.1	*Formica exsecta*	Yes	no	no
XP_029659523.1	*Formica exsecta*	no	Yes	Yes
XP_029662129.1	*Formica exsecta*	Yes	Yes	Yes
XP_029663041.1	*Formica exsecta*	Yes	Yes	no
XP_029664109.1	*Formica exsecta*	no	Yes	no
XP_029666096.1	*Formica exsecta*	no	no	no
XP_029668279.1	*Formica exsecta*	Yes	no	no
XP_029668528.1	*Formica exsecta*	no	Yes	Yes
XP_029670993.1	*Formica exsecta*	no	Yes	Yes
XP_029672500.1	*Formica exsecta*	Yes	Yes	no
XP_029672911.1	*Formica exsecta*	no	Yes	Yes
XP_029674039.1	*Formica exsecta*	Yes	no	no
XP_029674814.1	*Formica exsecta*	no	Yes	Yes
XP_029675459.1	*Formica exsecta*	no	Yes	Yes
XP_029678523.1	*Formica exsecta*	no	Yes	no
XP_029679398.1	*Formica exsecta*	no	Yes	Yes
XP_029679910.1	*Formica exsecta*	no	Yes	no
XP_029680138.1	*Formica exsecta*	no	no	no
XP_029680200.1	*Formica exsecta*	Yes	no	no
Ffu2_g02036_i1	*Formica fusca*	no	Yes	Yes
Ffu2_g06619_i1	*Formica fusca*	no	Yes	Yes
Ffu2_g07947_i2	*Formica fusca*	no	Yes	Yes
Ffu2_g10942_i1	*Formica fusca*	no	Yes	no
Ffu2_g11677_i1	*Formica fusca*	no	Yes	Yes
Ffu2_g11725_i1	*Formica fusca*	no	Yes	Yes
Ffu2_g12384_i1	*Formica fusca*	no	Yes	Yes
Ffu2_g13839_i1	*Formica fusca*	no	no	no
Fjap_g00631_i1	*Formica japonica*	no	Yes	Yes
Fjap_g01849_i1	*Formica japonica*	Yes	no	no
Fjap_g01929_i1	*Formica japonica*	no	Yes	Yes

Fjap_g01930_i1	*Formica japonica*	no	Yes	Yes
Fjap_g01977_i4	*Formica japonica*	Yes	Yes	Yes
Fjap_g03368_i1	*Formica japonica*	no	Yes	Yes
Fjap_g03376_i1	*Formica japonica*	no	Yes	Yes
Fjap_g04597_i1	*Formica japonica*	no	Yes	Yes
Fjap_g05008_i1	*Formica japonica*	no	Yes	Yes
Fjap_g05076_i1	*Formica japonica*	no	Yes	Yes
Fjap_g07598_i1	*Formica japonica*	no	Yes	Yes
Fjap_g07605_i1	*Formica japonica*	no	Yes	Yes
Fjap_g07614_i1	*Formica japonica*	Yes	Yes	Yes
Fjap_g08512_i1	*Formica japonica*	no	Yes	Yes
Fjap_g08788_i1	*Formica japonica*	no	Yes	Yes
Fjap_g09189_i1	*Formica japonica*	no	Yes	Yes
Fjap_g10250_i1	*Formica japonica*	no	Yes	Yes
Fjap_g12182_i1	*Formica japonica*	no	Yes	Yes
Fjap_g12262_i1	*Formica japonica*	no	Yes	no
Fjap_g12564_i1	*Formica japonica*	Yes	Yes	Yes
Fjap_g12566_i1	*Formica japonica*	Yes	Yes	Yes
Fjap_g12773_i1	*Formica japonica*	Yes	Yes	Yes
Fjap_g12912_i1	*Formica japonica*	no	Yes	Yes
Fjap_g12924_i1	*Formica japonica*	no	Yes	Yes
Fjap_g13125_i1	*Formica japonica*	no	Yes	Yes
Fjap_g13126_i1	*Formica japonica*	no	Yes	no
Fjap_g13128_i1	*Formica japonica*	no	Yes	Yes
Fjap_g13178_i1	*Formica japonica*	no	Yes	Yes
Fjap_g13849_i1	*Formica japonica*	no	Yes	Yes
Fjap_g13897_i1	*Formica japonica*	no	Yes	Yes
Fjap_g13900_i1	*Formica japonica*	no	Yes	Yes
Fjap_g13927_i1	*Formica japonica*	Yes	Yes	Yes
Fjap_g13928_i1	*Formica japonica*	Yes	Yes	Yes
Fjap_g14755_i2	*Formica japonica*	Yes	Yes	Yes
Fjap_g15257_i1	*Formica japonica*	no	Yes	Yes
Fjap_g15376_i1	*Formica japonica*	no	Yes	Yes
Fjap_g15393_i1	*Formica japonica*	no	Yes	Yes
Fjap_g15428_i1	*Formica japonica*	no	Yes	Yes
Fjap_g15618_i1	*Formica japonica*	no	Yes	Yes
Fjap_g16121_i1	*Formica japonica*	Yes	Yes	Yes
Fjap_g16137_i1	*Formica japonica*	no	Yes	no
Fjap_g16175_i1	*Formica japonica*	Yes	Yes	Yes
Fjap_g16779_i1	*Formica japonica*	no	Yes	no
Fjap_g16780_i1	*Formica japonica*	no	Yes	no
Fjap_g16928_i1	*Formica japonica*	Yes	Yes	Yes
Fjap_g16934_i1	*Formica japonica*	Yes	Yes	Yes
Fjap_g16955_i1	*Formica japonica*	Yes	Yes	no
Fjap_g17245_i1	*Formica japonica*	no	Yes	Yes
Fjap_g17247_i1	*Formica japonica*	no	Yes	no
Fjap_g17249_i1	*Formica japonica*	no	Yes	Yes
Fjap_g17583_i1	*Formica japonica*	no	Yes	Yes
Fjap_g18781_i1	*Formica japonica*	Yes	Yes	Yes
Fjap_g18809_i1	*Formica japonica*	Yes	Yes	Yes
Fjap_g18814_i1	*Formica japonica*	no	Yes	no
Fjap_g19340_i1	*Formica japonica*	Yes	Yes	Yes
Fsan_g00637_i1	*Formica sanguinea*	Yes	Yes	Yes
Fsan_g04314_i1	*Formica sanguinea*	no	Yes	Yes
Fsan_g04384_i1	*Formica sanguinea*	no	Yes	Yes
Fsan_g04845_i1	*Formica sanguinea*	no	Yes	Yes

Fsan_g04846_i1	*Formica sanguinea*	no	Yes	Yes
Fsan_g04961_i1	*Formica sanguinea*	no	Yes	Yes
Fsan_g04968_i1	*Formica sanguinea*	no	Yes	Yes
Fsan_g04980_i1	*Formica sanguinea*	no	Yes	Yes
Fsan_g08813_i1	*Formica sanguinea*	no	Yes	Yes
Fsan_g08828_i1	*Formica sanguinea*	no	Yes	Yes
Fsan_g08850_i1	*Formica sanguinea*	no	Yes	Yes
Fsan_g09692_i1	*Formica sanguinea*	no	Yes	Yes
Fsan_g12157_i1	*Formica sanguinea*	no	Yes	Yes
Fsan_g12990_i1	*Formica sanguinea*	no	Yes	Yes
Fsan_g13347_i1	*Formica sanguinea*	no	Yes	no
Fsan_g15309_i1	*Formica sanguinea*	no	Yes	no
Fsan_g16348_i1	*Formica sanguinea*	no	Yes	Yes
XP_011150999.1	*Harpegnathos saltator*	no	Yes	Yes
Isub_g00081_i1	*Iberoformica subrufa*	no	Yes	Yes
Isub_g08443_i1	*Iberoformica subrufa*	no	Yes	Yes
Isub_g09449_i1	*Iberoformica subrufa*	no	Yes	Yes
Isub_g11297_i1	*Iberoformica subrufa*	no	Yes	Yes
Isub_g12901_i1	*Iberoformica subrufa*	no	Yes	Yes
Isub_g12918_i1	*Iberoformica subrufa*	no	Yes	no
Isub_g14226_i1	*Iberoformica subrufa*	no	Yes	Yes
XP_011331792.1	*Ooceraea biroi*	no	Yes	Yes
XP_025073434.1	*Pogonomyrmex barbatus*	no	Yes	Yes
XP_011156366.1	*Solenopsis invicta*	no	Yes	Yes

*Protein domains identified by InterProScan

Table S4. Origin of biological samples used to generate genomic data

Sample ID	Species	Project	Social form	Supergene genotype	Location*	Country	Biosample accession	SRA accession	Trimmed reads	Mapped reads	Mapping percentage	Coverage (X)
DE90_pool_M	*F. selysi*	Assembly	Monogynous	M (male pool)	Derborence	Switzerland	SAMN53044953	SRR35923081	NA	NA	NA	NA
FI43_pool_P	*F. selysi*	Assembly	Polygynous	P (male pool)	Finges	Switzerland	SAMN53044954	SRR35923080	NA	NA	NA	NA
FsiM_HiC	*F. selysi*	Assembly	Monogynous	M (male pool)	Derborence	Switzerland	SAMN53044955	SRR35923079	NA	NA	NA	NA
FsiP_HiC	*F. selysi*	Assembly	Polygynous	P (male pool)	Derborence	Switzerland	SAMN53044956	SRR35923078	NA	NA	NA	NA
De434W1	*F. selysi*	Reseq	Monogynous	MM (worker)	Derborence	Switzerland	SAMN52853031	SRR35832584	53359646	52576208	98.53	19.5
De44W1	*F. selysi*	Reseq	Monogynous	MM (worker)	Derborence	Switzerland	SAMN52853033	SRR35832582	51505906	50889350	98.80	18.8
De67W1	*F. selysi*	Reseq	Monogynous	MM (worker)	Derborence	Switzerland	SAMN52853034	SRR35832580	50395218	49703640	98.63	18.4
De84W1	*F. selysi*	Reseq	Monogynous	MM (worker)	Derborence	Switzerland	SAMN52853035	SRR35832579	69336257	68591913	98.93	25.4
De115W1	*F. selysi*	Reseq	Monogynous	MM (worker)	Derborence	Switzerland	SAMN52853021	SRR35832595	47881204	47302166	98.79	17.5
De125W1	*F. selysi*	Reseq	Monogynous	MM (worker)	Derborence	Switzerland	SAMN52853022	SRR35832594	29810562	28448719	95.43	10.5
De172W1	*F. selysi*	Reseq	Monogynous	MM (worker)	Derborence	Switzerland	SAMN52853023	SRR35832593	56866990	55070308	96.84	20.4
De397W1	*F. selysi*	Reseq	Monogynous	MM (worker)	Derborence	Switzerland	SAMN52853030	SRR35832585	39941369	37729514	94.46	14.0
674W1	*F. selysi*	Reseq	Monogynous	MM (worker)	Finges	Switzerland	SAMN52853006	SRR35832592	55332734	55269077	99.88	20.5
677W1	*F. selysi*	Reseq	Monogynous	MM (worker)	Finges	Switzerland	SAMN52853007	SRR35832581	52682877	52616583	99.87	19.5
700W3	*F. selysi*	Reseq	Monogynous	MM (worker)	Finges	Switzerland	SAMN52853008	SRR35832570	51198078	51112547	99.83	18.9
701W1	*F. selysi*	Reseq	Monogynous	MM (worker)	Finges	Switzerland	SAMN52853009	SRR35832561	60747214	60520267	99.63	22.4
LB1W2	*F. selysi*	Reseq	Monogynous	MM (worker)	Les Bussets	France	SAMN52853036	SRR35832578	60459722	60348159	99.82	22.4
LB2W2	*F. selysi*	Reseq	Monogynous	MM (worker)	Les Bussets	France	SAMN52853037	SRR35832577	67342228	67217415	99.81	24.9
LB3W2	*F. selysi*	Reseq	Monogynous	MM (worker)	Les Bussets	France	SAMN52853038	SRR35832576	60921453	60772419	99.76	22.5
LB4W2	*F. selysi*	Reseq	Monogynous	MM (worker)	Les Bussets	France	SAMN52853039	SRR35832575	60681987	60551164	99.78	22.4
LB7W2	*F. selysi*	Reseq	Monogynous	MM (worker)	Les Bussets	France	SAMN52853042	SRR35832572	60492356	60352068	99.77	22.4
Sal1W1	*F. selysi*	Reseq	Monogynous	MM (worker)	Sallanches	France	SAMN52853045	SRR35832569	64505735	64206526	99.54	23.8
Sal2W1	*F. selysi*	Reseq	Monogynous	MM (worker)	Sallanches	France	SAMN52853046	SRR35832568	63590144	63336823	99.60	23.5
Sal3W1	*F. selysi*	Reseq	Monogynous	MM (worker)	Sallanches	France	SAMN52853047	SRR35832567	64152400	63977614	99.73	23.7
Sal4W1	*F. selysi*	Reseq	Monogynous	MM (worker)	Sallanches	France	SAMN52853048	SRR35832566	60382115	60221210	99.73	22.3
De107W1	*F. selysi*	Reseq	Polygynous	MP (worker)	Derborence	Switzerland	SAMN52853020	SRR35832596	50010529	49764063	99.51	18.4
De193W1	*F. selysi*	Reseq	Polygynous	MP (worker)	Derborence	Switzerland	SAMN52853024	SRR35832591	61204328	61061651	99.77	22.6
De254W1	*F. selysi*	Reseq	Polygynous	MP (worker)	Derborence	Switzerland	SAMN52853025	SRR35832590	98636497	98157997	99.51	36.4
De287W1	*F. selysi*	Reseq	Polygynous	MP (worker)	Derborence	Switzerland	SAMN52853028	SRR35832587	48734282	48522297	99.57	18.0
174W1	*F. selysi*	Reseq	Polygynous	MP (worker)	Finges	Switzerland	SAMN52853004	SRR35832604	46080632	46026116	99.88	17.0
LB5W2	*F. selysi*	Reseq	Polygynous	MP (worker)	Les_Bussets	France	SAMN52853040	SRR35832574	57592102	56894477	98.79	21.1
LB6W2	*F. selysi*	Reseq	Polygynous	MP (worker)	Les_Bussets	France	SAMN52853041	SRR35832573	59324452	58658575	98.88	21.7
LB8W2	*F. selysi*	Reseq	Polygynous	MP (worker)	Les_Bussets	France	SAMN52853043	SRR35832571	75410605	74842457	99.25	27.7
Sal10W6	*F. selysi*	Reseq	Polygynous	MP (worker)	Sallanches	France	SAMN52853044	SRR35832562	47596951	46824392	98.38	17.3
Sal5W3	*F. selysi*	Reseq	Polygynous	MP (worker)	Sallanches	France	SAMN52853049	SRR35832565	62315145	62228501	99.86	23.0

Sal6W5	*F. selysi*	Reseq	Polygynous	MP (worker)	Sallanches	France	SAMN52853050	SRR35832564	52009019	39589893	76.12	14.7
Sal9W4	*F. selysi*	Reseq	Polygynous	MP (worker)	Sallanches	France	SAMN52853051	SRR35832563	65249358	65148776	99.85	24.1
De259W2	*F. selysi*	Reseq	Polygynous	PP (worker)	Derborence	Switzerland	SAMN52853026	SRR35832589	40684304	40382563	99.26	15.0
De267W1	*F. selysi*	Reseq	Polygynous	PP (worker)	Derborence	Switzerland	SAMN52853027	SRR35832588	55709616	55485637	99.60	20.6
De299W1	*F. selysi*	Reseq	Polygynous	PP (worker)	Derborence	Switzerland	SAMN52853029	SRR35832586	51873484	51716554	99.70	19.2
De446W2	*F. selysi*	Reseq	Polygynous	PP (worker)	Derborence	Switzerland	SAMN52853032	SRR35832583	57422533	55907831	97.36	20.7
508W1	*F. selysi*	Reseq	Polygynous	PP (worker)	Finges	Switzerland	SAMN52853005	SRR35832603	55171715	55076133	99.83	20.4
703W4	*F. selysi*	Reseq	Polygynous	PP (worker)	Finges	Switzerland	SAMN52853010	SRR35832560	50067871	50001343	99.87	18.5
706W4	*F. selysi*	Reseq	Polygynous	PP (worker)	Finges	Switzerland	SAMN52853011	SRR35832559	50569956	50469016	99.80	18.7
710W2	*F. selysi*	Reseq	Polygynous	PP (worker)	Finges	Switzerland	SAMN52853012	SRR35832558	56949590	56789825	99.72	21.0
713W3	*F. selysi*	Reseq	Polygynous	PP (worker)	Finges	Switzerland	SAMN52853013	SRR35832557	50528215	50310034	99.57	18.6
715W2	*F. selysi*	Reseq	Polygynous	PP (worker)	Finges	Switzerland	SAMN52853014	SRR35832602	63792821	63597210	99.69	23.6
716W1	*F. selysi*	Reseq	Polygynous	PP (worker)	Finges	Switzerland	SAMN52853015	SRR35832601	50160694	48612033	96.91	18.0
722W1	*F. selysi*	Reseq	Polygynous	PP (worker)	Finges	Switzerland	SAMN52853016	SRR35832600	52415008	52268952	99.72	19.4
733W1	*F. selysi*	Reseq	Polygynous	PP (worker)	Finges	Switzerland	SAMN52853017	SRR35832599	49788576	49717526	99.86	18.4
748W1	*F. selysi*	Reseq	Polygynous	PP (worker)	Finges	Switzerland	SAMN52853018	SRR35832598	62481686	62359933	99.81	23.1
750W2	*F. selysi*	Reseq	Polygynous	PP (worker)	Finges	Switzerland	SAMN52853019	SRR35832597	61932191	61805898	99.80	22.9

*GPS coordinates of the different locations: Derborence, 46.286341°N, 7.225548°E; Finges, 46.311984°N, 7.605539°E; Les Bussets, 44.15095°N, 5.43077°E; Sallanches: 45.57487°N, 6.37540°E

Table S5. Origin of biological samples used to generate transcriptomic data

Sample ID	Species	Caste	Tissue	Social form	Supergene genotype	Location*	Country	Biosample accession	SRA accession	Trimmed reads	Pseudoaligned reads	% pseudoaligned
103s	*F. selysi*	Virgin queen	head+thorax	Polygynous	MP	Finges	Switzerland	SAMN52899701	SRR35860623	27656127	15133941	54.7
110s	*F. selysi*	Virgin queen	head+thorax	Polygynous	MP	Finges	Switzerland	SAMN52899702	SRR35860622	28512867	17962214	63.0
113s	*F. selysi*	Virgin queen	head+thorax	Polygynous	PP	Finges	Switzerland	SAMN52899703	SRR35860611	28049319	17458240	62.2
132s	*F. selysi*	Worker	head+thorax	Polygynous	MP	Finges	Switzerland	SAMN52899704	SRR35860600	26766322	15353774	57.4
147s	*F. selysi*	Mated queen	head+thorax	Polygynous	PP	Finges	Switzerland	SAMN52899705	SRR35860589	24059473	16182465	67.3
52s	*F. selysi*	Virgin queen	head+thorax	Polygynous	MP	Finges	Switzerland	SAMN52899706	SRR35860578	26333091	14802260	56.2
59s	*F. selysi*	Virgin queen	head+thorax	Polygynous	PP	Finges	Switzerland	SAMN52899707	SRR35860568	28923242	18688856	64.6
5A1	*F. selysi*	Worker	head+thorax	Monogynous	MM	Finges	Switzerland	SAMN52899708	SRR35860567	43130419	31442454	72.9
5A4	*F. selysi*	Worker	head+thorax	Monogynous	MM	Finges	Switzerland	SAMN52899709	SRR35860566	35147389	25968386	73.9
61s	*F. selysi*	Mated queen	head+thorax	Polygynous	PP	Finges	Switzerland	SAMN52899710	SRR35860565	22460776	15129224	67.4
64s	*F. selysi*	Mated queen	head+thorax	Polygynous	PP	Finges	Switzerland	SAMN52899711	SRR35860621	20893548	13061267	62.5
65s	*F. selysi*	Mated queen	head+thorax	Polygynous	MP	Finges	Switzerland	SAMN52899712	SRR35860620	24353954	12787113	52.5
72s	*F. selysi*	Worker	head+thorax	Polygynous	PP	Finges	Switzerland	SAMN52899713	SRR35860619	21422294	11763540	54.9
74s	*F. selysi*	Worker	head+thorax	Polygynous	MP	Finges	Switzerland	SAMN52899714	SRR35860618	21676607	12363242	57.0
80s	*F. selysi*	Worker	head+thorax	Polygynous	PP	Finges	Switzerland	SAMN52899715	SRR35860617	28323140	18967740	67.0
81s	*F. selysi*	Worker	head+thorax	Polygynous	MP	Finges	Switzerland	SAMN52899716	SRR35860616	21110985	12296766	58.2
82s	*F. selysi*	Worker	head+thorax	Polygynous	PP	Finges	Switzerland	SAMN52899717	SRR35860615	23863974	15933269	66.8
86s	*F. selysi*	Virgin queen	head+thorax	Polygynous	MP	Finges	Switzerland	SAMN52899718	SRR35860614	25054754	11493546	45.9
87s	*F. selysi*	Virgin queen	head+thorax	Polygynous	PP	Finges	Switzerland	SAMN52899719	SRR35860613	29651720	15873886	53.5
90s	*F. selysi*	Virgin queen	head+thorax	Polygynous	MP	Finges	Switzerland	SAMN52899720	SRR35860612	25542973	15516838	60.7
99s	*F. selysi*	Mated queen	head+thorax	Polygynous	MP	Finges	Switzerland	SAMN52899721	SRR35860610	22218484	15246795	68.6
A_VQ	*F. selysi*	Virgin queen	head+thorax	Monogynous	MM	Finges	Switzerland	SAMN52899722	SRR35860609	27394920	18833012	68.7
B_VQ	*F. selysi*	Virgin queen	head+thorax	Monogynous	MM	Finges	Switzerland	SAMN52899723	SRR35860608	32102145	25035578	78.0
C_VQ	*F. selysi*	Virgin queen	head+thorax	Monogynous	MM	Finges	Switzerland	SAMN52899724	SRR35860607	29937596	20813144	69.5
D_W	*F. selysi*	Worker	head+thorax	Polygynous	MP	Finges	Switzerland	SAMN52899725	SRR35860606	28800559	20941198	72.7
E_W	*F. selysi*	Worker	head+thorax	Polygynous	MP	Finges	Switzerland	SAMN52899726	SRR35860605	29270169	18768221	64.1
F_W	*F. selysi*	Worker	head+thorax	Polygynous	MP	Finges	Switzerland	SAMN52899727	SRR35860604	25472905	16025381	62.9
G_VQ	*F. selysi*	Virgin queen	head+thorax	Monogynous	MM	Finges	Switzerland	SAMN52899728	SRR35860603	28963483	20689100	71.4
H_VQ	*F. selysi*	Virgin queen	head+thorax	Polygynous	PP	Finges	Switzerland	SAMN52899729	SRR35860602	29959959	13205754	44.1
I_VQ	*F. selysi*	Virgin queen	head+thorax	Polygynous	PP	Finges	Switzerland	SAMN52899730	SRR35860601	28702243	17692543	61.6
J_VQ	*F. selysi*	Virgin queen	head+thorax	Monogynous	MM	Finges	Switzerland	SAMN52899731	SRR35860599	26993350	16991535	62.9
K_VQ	*F. selysi*	Virgin queen	head+thorax	Polygynous	PP	Finges	Switzerland	SAMN52899732	SRR35860598	28824728	18226585	63.2
L_MQ	*F. selysi*	Mated queen	head+thorax	Polygynous	MP	Finges	Switzerland	SAMN52899733	SRR35860597	25433502	18344511	72.1
M_MQ	*F. selysi*	Mated queen	head+thorax	Monogynous	MM	Finges	Switzerland	SAMN52899734	SRR35860596	25114187	18023687	71.8
N_MQ	*F. selysi*	Mated queen	head+thorax	Monogynous	MM	Finges	Switzerland	SAMN52899735	SRR35860595	27876580	19917968	71.5
O_MQ	*F. selysi*	Mated queen	head+thorax	Monogynous	MM	Finges	Switzerland	SAMN52899736	SRR35860594	22939688	16348847	71.3
P_MQ	*F. selysi*	Mated queen	head+thorax	Polygynous	MP	Finges	Switzerland	SAMN52899737	SRR35860593	24420057	17784854	72.8
Pct11	*F. selysi*	Worker	head+thorax	Polygynous	MP	Finges	Switzerland	SAMN52899738	SRR35860592	33337715	23512267	70.5

Pct16	*F. selysi*	Worker	head+thorax	Monogynous	MM	Finges	Switzerland	SAMN52899739	SRR35860591	28100396	18608934	66.2
Pct17	*F. selysi*	Worker	head+thorax	Monogynous	MM	Finges	Switzerland	SAMN52899740	SRR35860590	33721819	21756192	64.5
Pct18	*F. selysi*	Worker	head+thorax	Polygynous	PP	Finges	Switzerland	SAMN52899741	SRR35860588	30113010	19539702	64.9
Pct19	*F. selysi*	Worker	head+thorax	Polygynous	PP	Finges	Switzerland	SAMN52899742	SRR35860587	35809017	22205882	62.0
Pct20	*F. selysi*	Worker	head+thorax	Polygynous	PP	Finges	Switzerland	SAMN52899743	SRR35860586	31203341	21875994	70.1
Q_6MQ	*F. selysi*	Mated queen	head+thorax	Polygynous	PP	Finges	Switzerland	SAMN52899744	SRR35860585	40239442	28849411	71.7
R_7MQ	*F. selysi*	Mated queen	head+thorax	Monogynous	MM	Finges	Switzerland	SAMN52899745	SRR35860584	25088769	18368084	73.2
S_683	*F. selysi*	Virgin queen	head+thorax	Monogynous	MM	Finges	Switzerland	SAMN52899746	SRR35860583	28842494	20314848	70.4
Sze1a	*F. selysi*	Mated queen	head+thorax	Polygynous	MP	Finges	Switzerland	SAMN52899747	SRR35860582	29852345	20434418	68.5
Sze1c	*F. selysi*	Mated queen	head+thorax	Polygynous	PP	Finges	Switzerland	SAMN52899748	SRR35860581	27807368	19371739	69.7
Sze1f	*F. selysi*	Mated queen	head+thorax	Polygynous	PP	Finges	Switzerland	SAMN52899749	SRR35860580	27913131	17195242	61.6
Sze1g	*F. selysi*	Mated queen	head+thorax	Monogynous	MM	Finges	Switzerland	SAMN52899750	SRR35860579	31570168	19160412	60.7
Sze1l	*F. selysi*	Mated queen	head+thorax	Polygynous	MP	Finges	Switzerland	SAMN52899751	SRR35860577	29209458	19621936	67.2
Sze1n	*F. selysi*	Worker	head+thorax	Monogynous	MM	Finges	Switzerland	SAMN52899752	SRR35860576	21466119	10466030	48.8
Sze1o	*F. selysi*	Worker	head+thorax	Monogynous	MM	Finges	Switzerland	SAMN52899753	SRR35860575	28444184	16932031	59.5
Sze1p	*F. selysi*	Worker	head+thorax	Polygynous	MP	Finges	Switzerland	SAMN52899754	SRR35860574	31998752	20629380	64.5
T_686	*F. selysi*	Virgin queen	head+thorax	Polygynous	MP	Finges	Switzerland	SAMN52899755	SRR35860573	32328698	18016593	55.7
U_683	*F. selysi*	Mated queen	head+thorax	Monogynous	MM	Finges	Switzerland	SAMN52899756	SRR35860572	27732176	20667798	74.5
W_600	*F. selysi*	Virgin queen	head+thorax	Monogynous	MM	Finges	Switzerland	SAMN52899757	SRR35860571	25406734	18348866	72.2
X_602	*F. selysi*	Virgin queen	head+thorax	Monogynous	MM	Finges	Switzerland	SAMN52899758	SRR35860570	25696167	16778417	65.3
Y_587	*F. selysi*	Virgin queen	head+thorax	Monogynous	MM	Finges	Switzerland	SAMN52899759	SRR35860569	30561832	19819579	64.9

*GPS coordinates: Finges, 46.311984°N, 7.605539°E

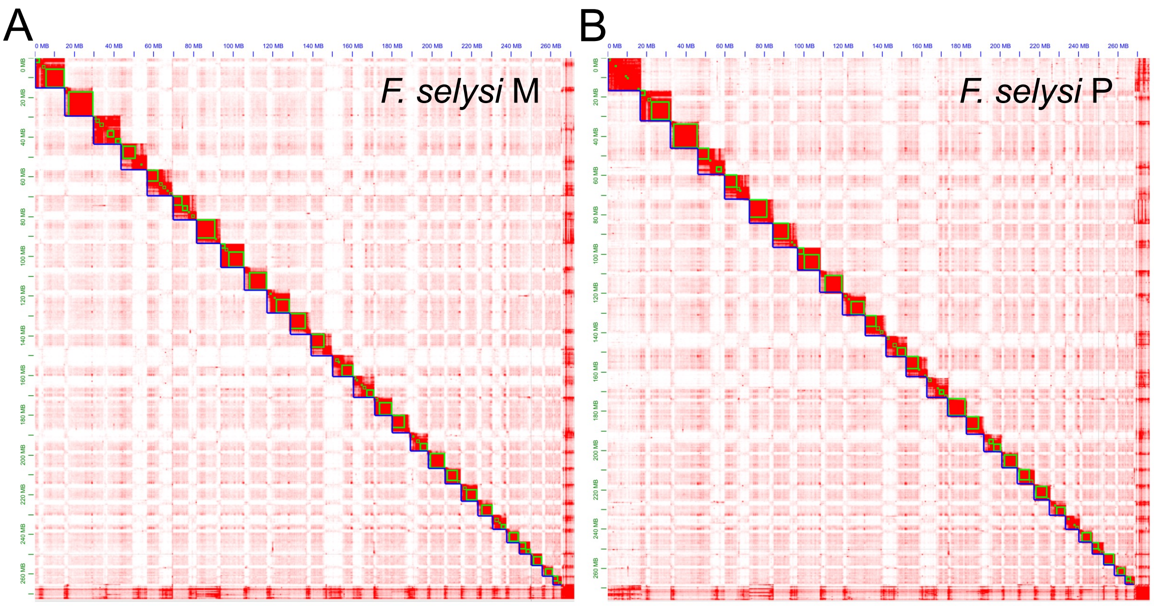

Figure S1. Heatmaps of HiC frequency contacts along (A) *Formica selysi* “monogynous” (M) and (B) *F. selysi* “polygynous” (P) genome assemblies. Blue lines show super scaffolds corresponding to the 27 chromosomes and green lines indicate contigs. Chromosomes are ordered by size for each social form with x and y axis showing genome cumulative size in megabase pairs.

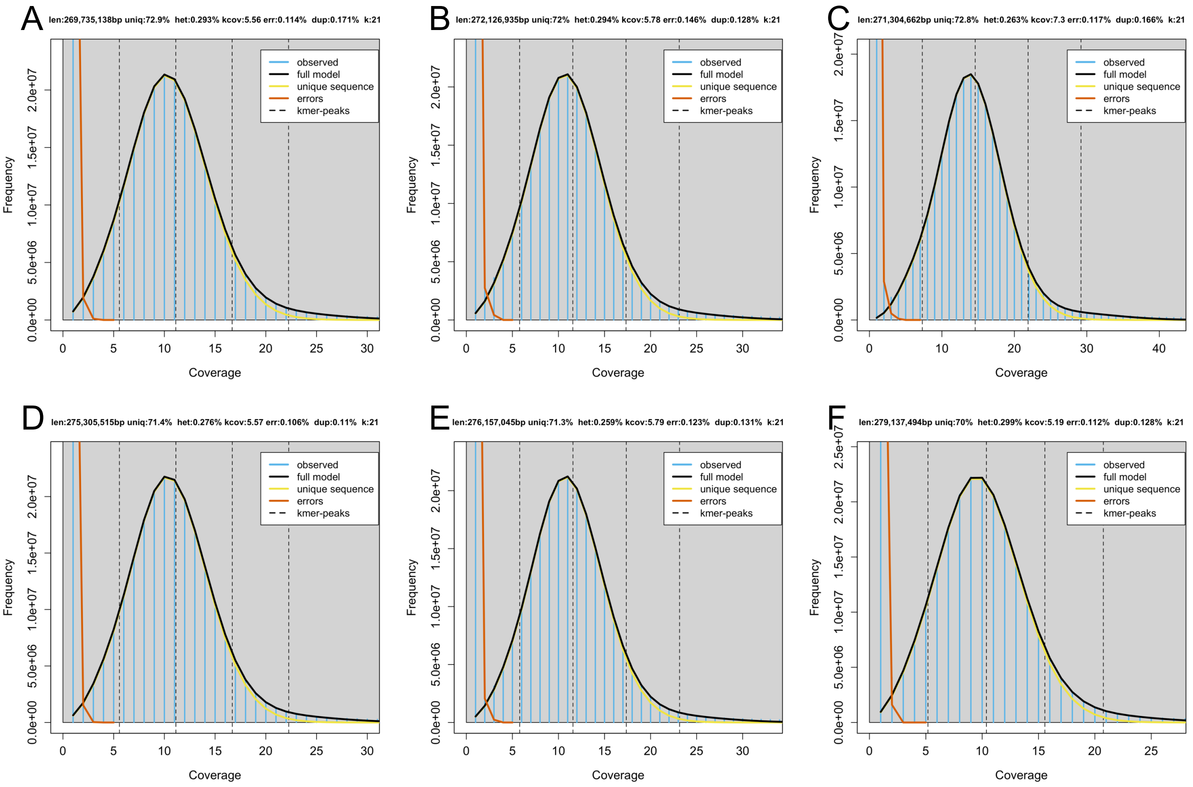

Figure S2. Genome size estimates based on k-mer profile analyses of paired-end Illumina short reads from (A-C) three *F. selysi* monogynous (*MM*) workers and (D-F) three *F. selysi* polygynous (*PP*) workers. Sample IDs: (A) De115W1; (B) De67W1; (C) De84W1; (D) 703W4; (E) 722W; (F) 733W1 (Supplementary table S1). len: estimated total length of the genome, uniq: percentage of the genome that is unique (not repetitive), het: heterozygosity rate, kcov: estimated k-mer coverage, err: sequencing error rate, dup: duplication rate, k: k-mer size.

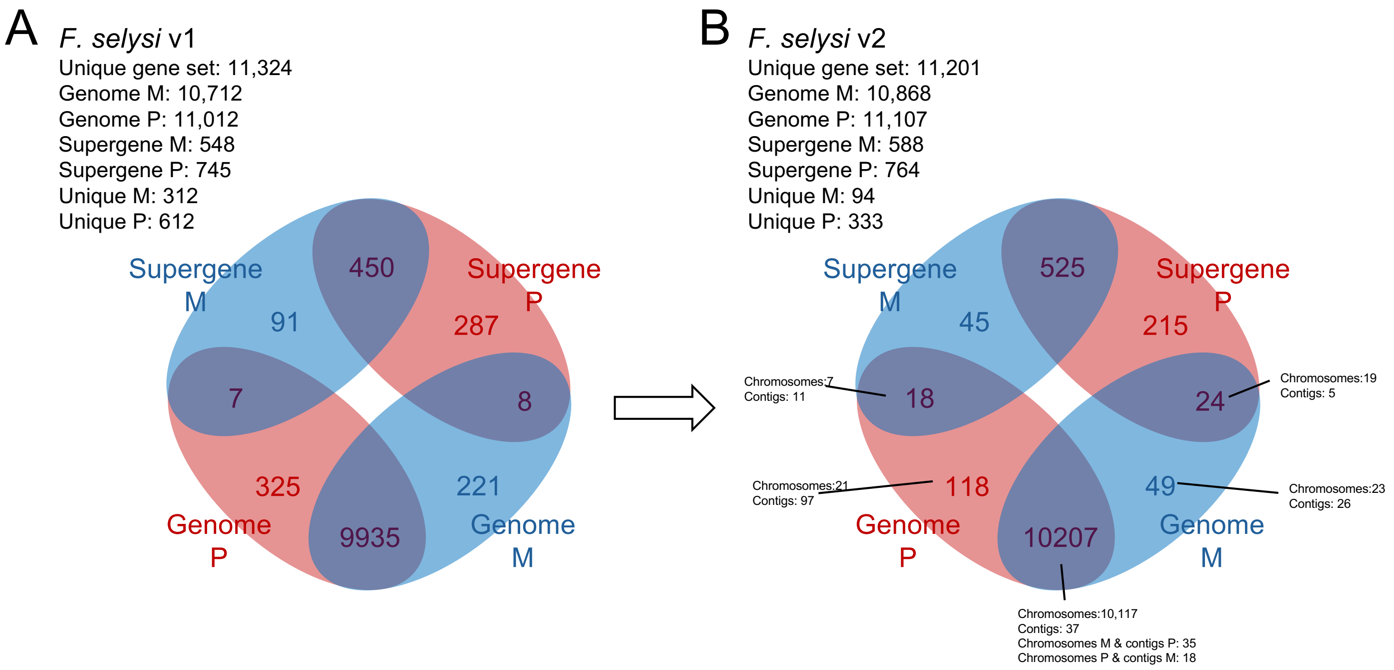

Figure S3. Annotation changes between (A) the first automatic annotation v1 and (B) the manually corrected annotation v2 of the *F. selysi* M and P genome assemblies.

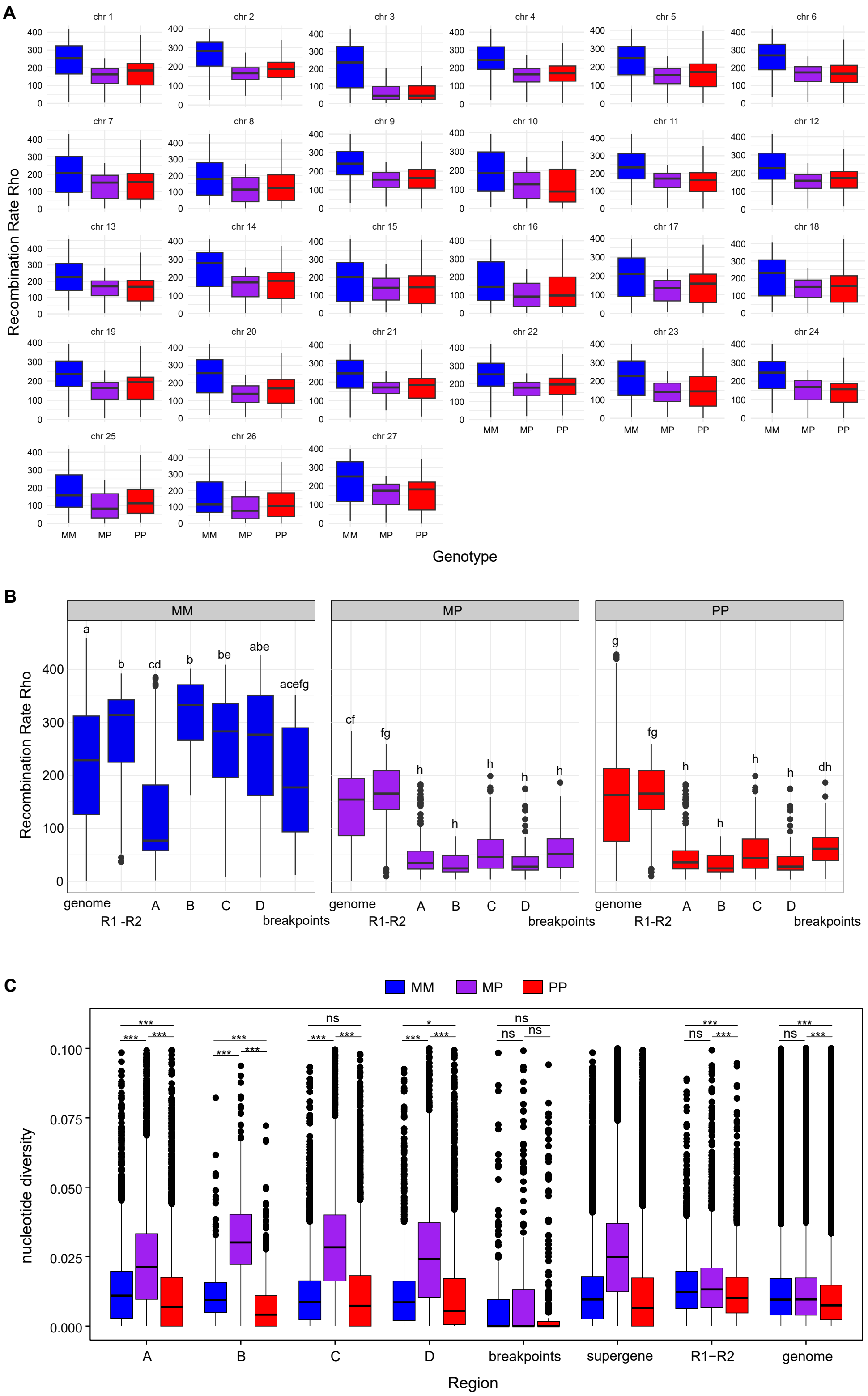

Figure S4. Recombination rate and nucleotide diversity calculated for each chromosome and region of the supergene for different social forms (workers: *MM*, n=21; *MP*, n=12; *PP*, n=15). (A) Recombination rates calculated in 100 kb windows with 20 kb overlaps for each chromosome show consistently higher in rates in *MM* compared to *MP* and *PP* individuals (workers). (B) Recombination rates for each region of the supergene and the rest of the genome are consistently lower for *MP* and *PP* individuals compared to *MM*. (C) Nucleotide diversity (pi) calculated in 1kb windows across supergene regions and the rest of the genome. *PP* has lower genetic diversity across nearly all regions of the genome compared to *MM* and *MP* individuals.

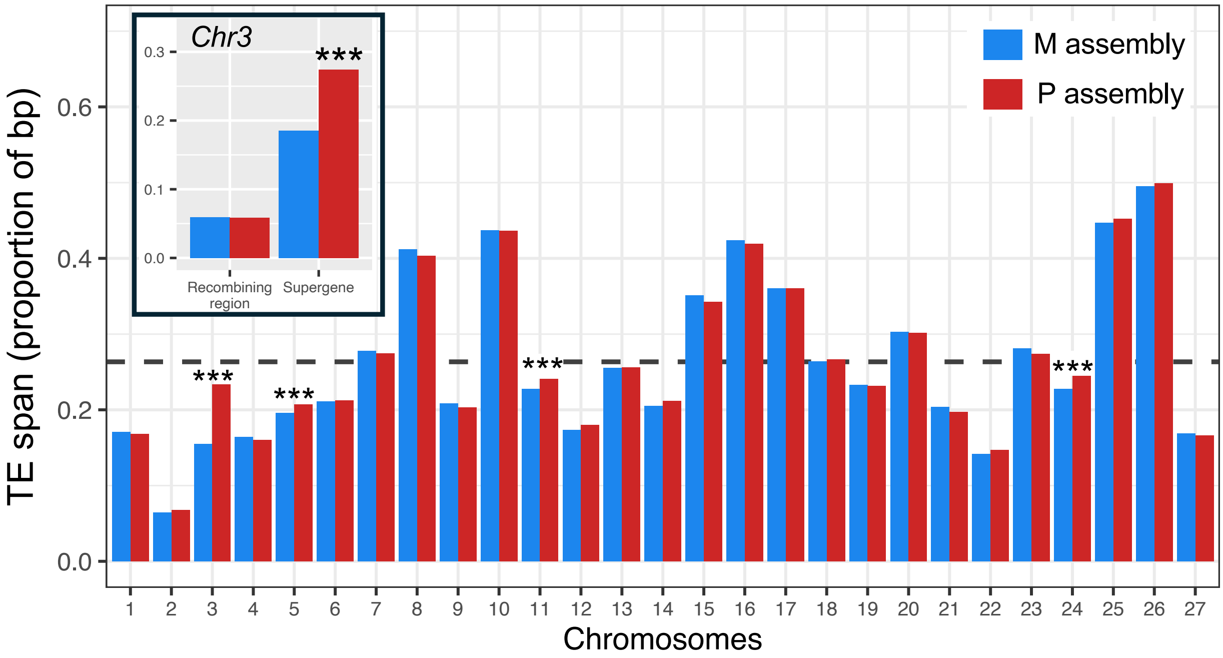

Figure S5. Transposable element (TE) span in chromosomes from *F. selysi* M and P genome assemblies. TE span ranges from 2% (chromosome 2) to 50% (chromosome 26) across the M and P genome assemblies. Significant TE span differences superior to 1% are observed between M and P for chromosomes 3, 5, 11 and 26 (proportion test with Bonferroni adjustment of *p*-values). The 8% differences between the two chromosomes 3 is due to a higher TE span in the *P* supergene haplotype as recombining regions of chromosome 3 do not differ in TE span between M and P assemblies. The dashed line indicates the average TE span across all chromosomes (26%). Statistical significance: ***p<0.001

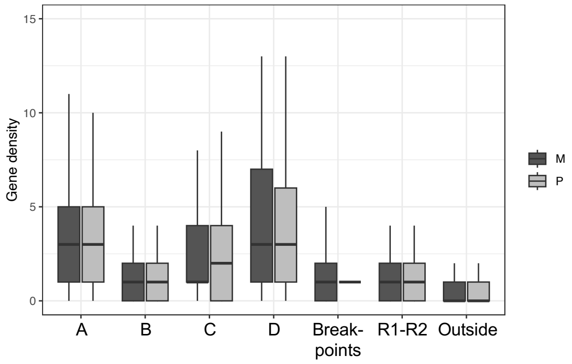

Figure S6. Comparison of gene densities over 50-kb fixed windows between the different regions of the chromosome 3 and the rest of the genome for the monogynous (M) and polygynous (P) social form. All comparisons between M and P were non-significant.

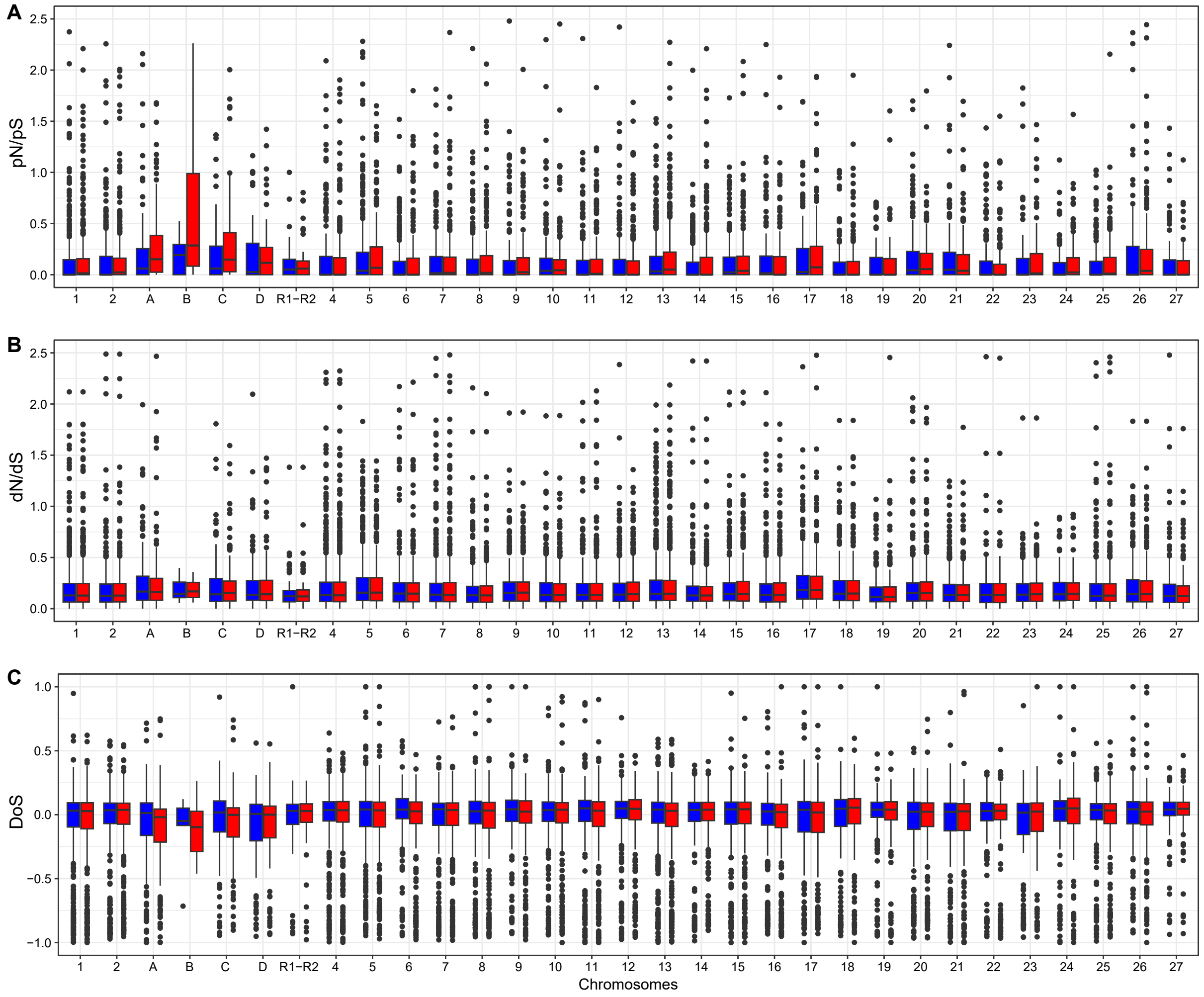

Figure S7. (A) Within species polymorphism (pN/pS), (B) dN/dS using *Polyergus* species as outgroup, and (C) direction of selection (DoS) calculated for 10,620 protein coding genes in the *F. selysi* genome. Each point corresponds to a protein-coding gene, grouped by chromosome and social form assembly. For chromosome 3, the collinear regions R1 and R2 (3R) and re-arranged regions (3A-D) have been represented separately. Region 3A shows higher pN/pS ratio, and lower DoS, and region 3C shows higher pN/pS ratio in the P genome assembly compared to the M genome assembly (Wilcoxon rank sum test).

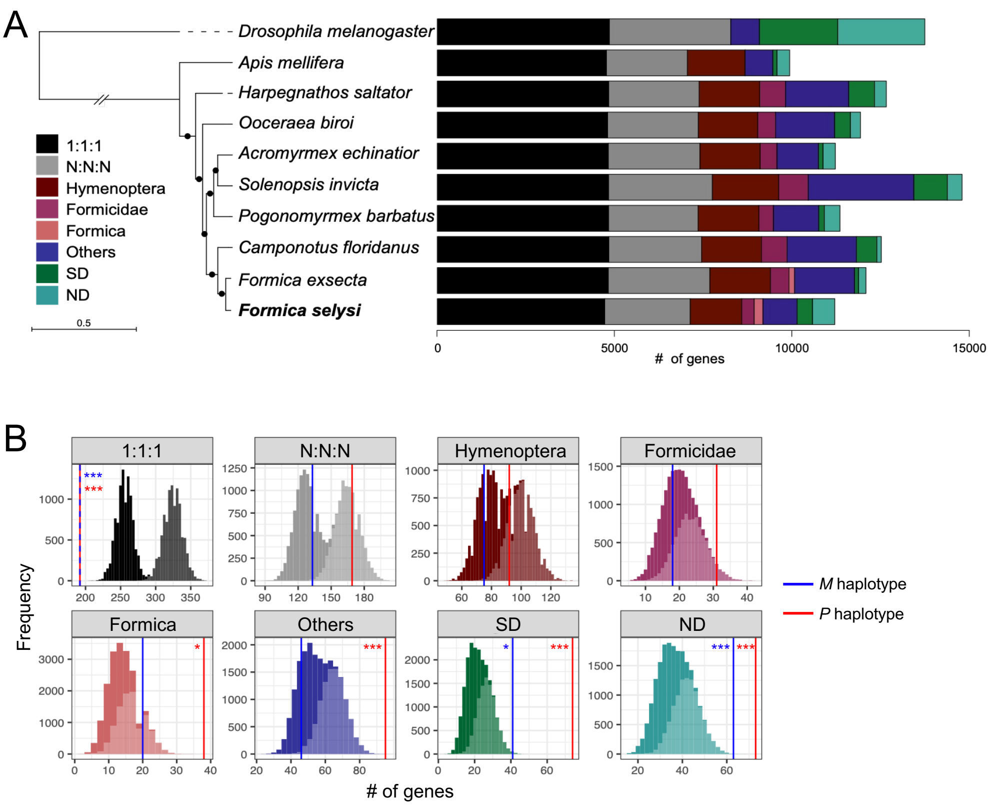

Figure S8. (A) Maximum-likelihood tree of the 10 insect species included in the analysis is based on 4,462 single-copy orthologues, with *Drosophila melanogaster* as outgroup. Black dots represent branches with bootstrap support of 100. The bar plots indicate orthology profiles of the 10 insect genomes. The orthologue categories are listed and color coded in the legend on the left. SD indicates species-specific duplicated genes, and ND species-specific single-copy genes. For each orthologue category, absence in one genome was allowed, except for the 1:1:1 category (for which absence in *D. melanogaster* was not allowed), and for the *Formica* category. (B) Enrichment of each orthologue category in each supergene haplotype. For each orthologue category, dark and pale color histograms show simulated distributions based on randomly resampling 588 and 764 genes for the *M* and *P* haplotypes, respectively, from the genome-wide distribution of orthologues in *F. selysi* (10,000 iterations). Blue and red vertical lines indicate observed gene numbers in this orthologue category for the *M* and *P* haplotypes, respectively.

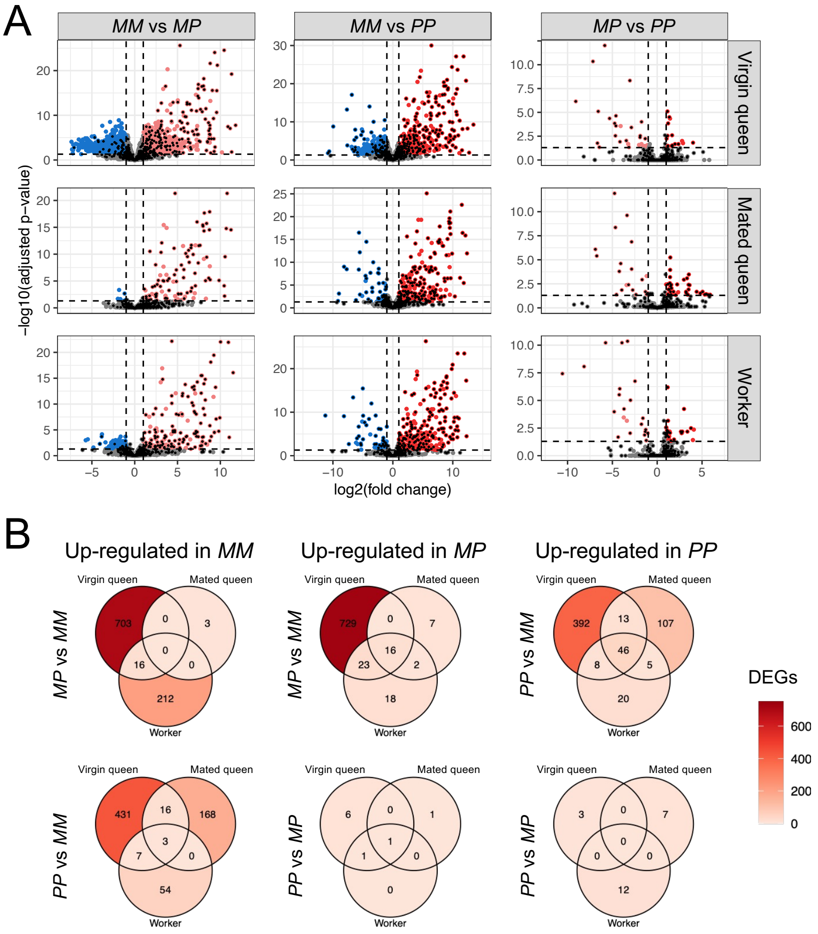

Figure S9. (A) Volcano plots illustrating differentially expressed genes (DEGs) between *F. selysi* virgin queens, mated queens, and workers from monogynous (homozygous *MM* at the supergene) and polygynous colonies (heterozygous *MP* and homozygous *PP* at the supergene). Genes up-regulated in *MM* (blue), *MP* (pink), and *PP* (red) individuals are shown. Black dots highlight genes located within the supergene. Genes are classified as DEGs when log2(fold-change) (LFC) ≥2 (horizontal dashed lines) and adjusted *p*-value ≤0.05 (vertical dashed lines). (B) Overlap of genotype-specific differentially expressed genes (DEGs) from outside the supergene across castes. *MM* individuals are from monogynous colonies while *MP* and *PP* individuals originate from polygynous colonies. Genes are classified as DEGs if they exhibit a fold-change (FC)≥2 and an adjusted *p*-value≤0.05.

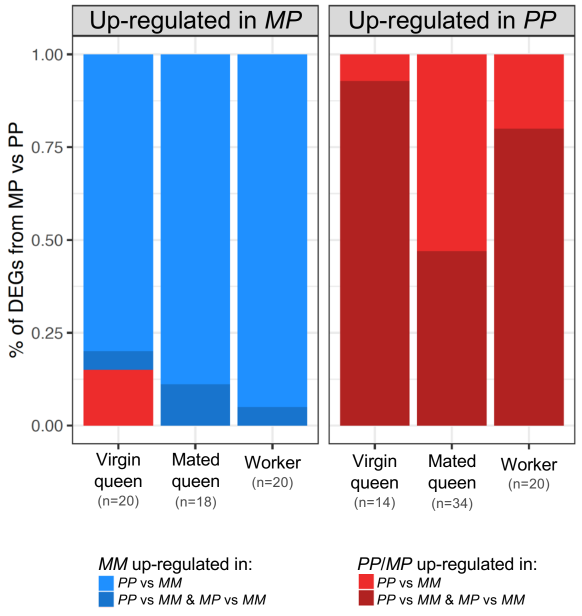

Figure S10. Supergene differentially expressed genes (DEGs) between *MP* and *PP* individuals from polygynous colonies. Across gyne, queen and worker castes, 29 and 56 genes are up-regulated in *MP* and *PP* individuals respectively when comparing both genotypes (non-redundant genes across all DEGs). Most of the *MP* up-regulated genes (left panel) are also up-regulated in *MM* individuals from monogynous colonies when compared to individuals from polygynous colonies (*MP* or *PP*), except in gynes where few discrepancies are observed. All the *PP* up-regulated genes (right panel) are also found up-regulated in *MP* or *PP* when compared to *MM* individuals from monogynous colonies.

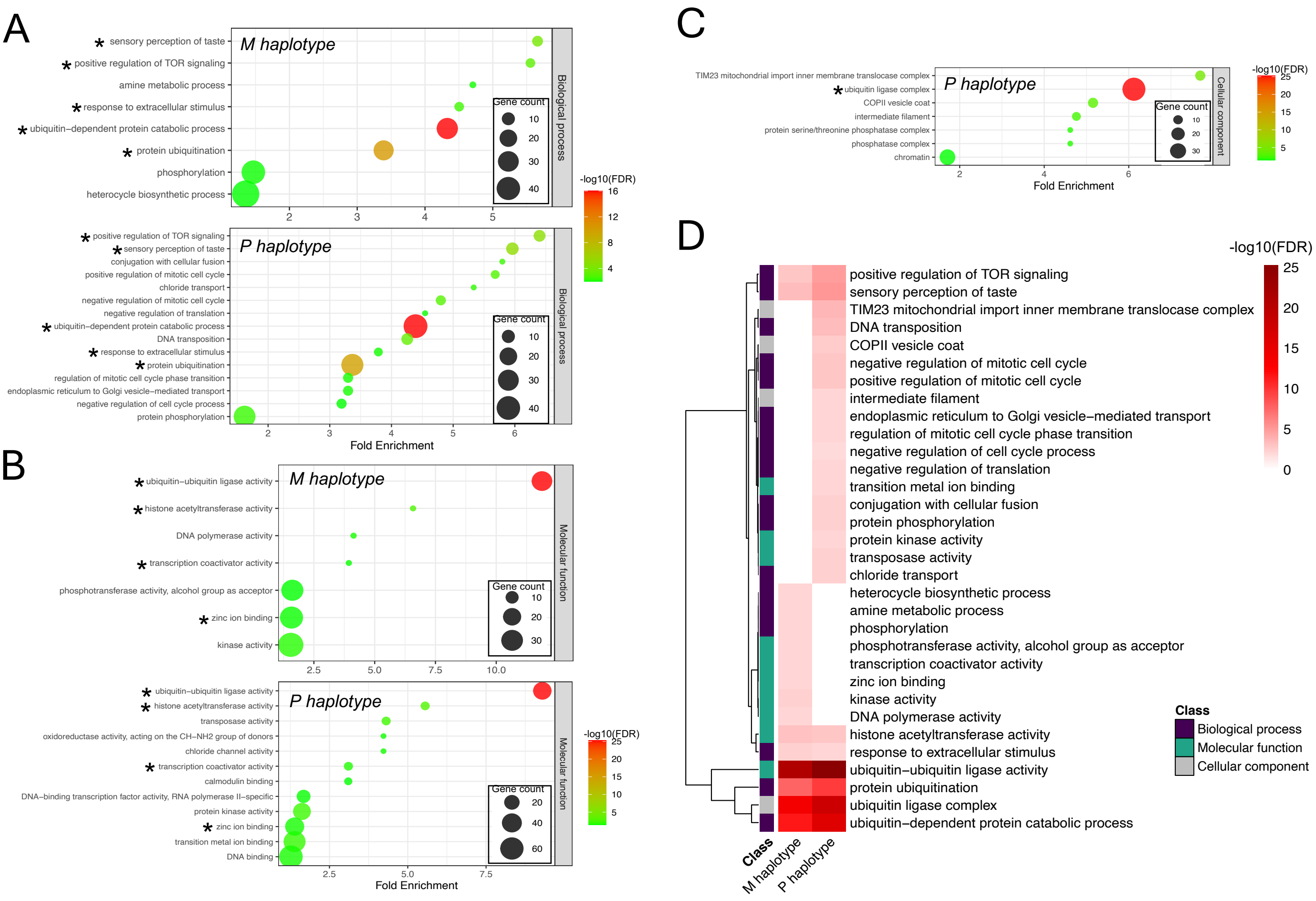

Figure S11. Gene Ontology (GO) enrichment in haplotypes of the supergene and monogynous- and polygynous-biased sets of differentially expressed genes (DEGs). The Blast2GO pipeline successfully assigned at least one GO term to 8,789 genes in the monogynous genome annotation and 8,956 genes in the polygynous genome annotation, covering 81% of the *F. selysi* gene set. The bubble plots present gene counts and fold enrichment for significantly enriched GO terms across three major categories: Biological Process (A), Molecular Function (B), and Cellular Component (C). Stars mark GO terms enriched in both *M* and *P* haplotypes, highlighting shared functional roles. The unique Cellular Component GO term enriched in the *M* haplotype is also marked by a star, as it overlaps with a GO term present in the *P* haplotype. (D) Hierarchical heatmaps clustering enriched GO terms in *F. selysi* supergene haplotypes based on False Discovery Rate (FDR), providing an overview of functional similarities and differences.

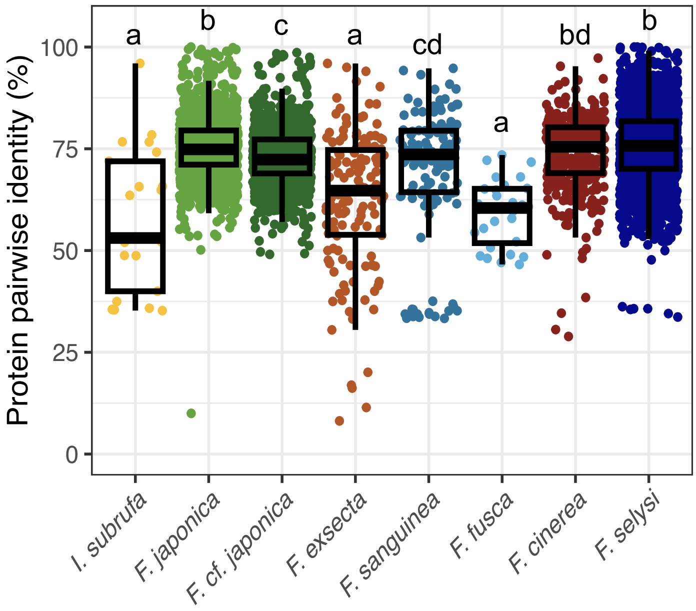

Figure S12. UBE4B protein similarity within *Formica* species. Percentages of amino-acid identity were extracted from the distance matrix generated by ClustalW v1.2.3 (Sievers and Higgins 2018) and only within species pairs were kept. Letters denote significant differences (Dunn's post-hoc test after a Kruskal–Wallis, c^2=268.3, df=7, p<0.001).

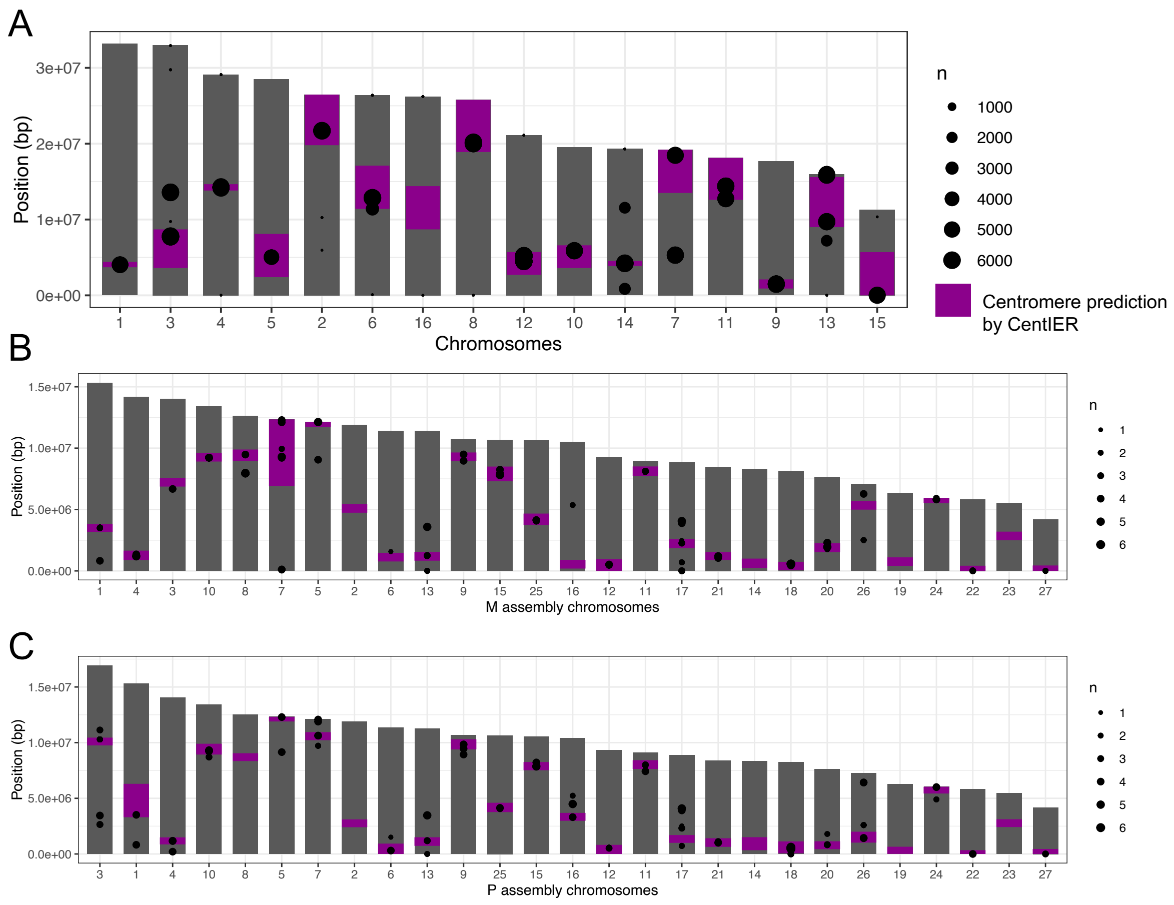

Figure S13. Centromere prediction in *Solenopsis invicta* chromosomes (A) and *Formica selysi* M (B) and P (C) assembly chromosomes. CentIER centromere prediction is shown in purple and black circles indicate satellite DNA match counts (mapping by blastn). On average *S. invicta* CentIER predicted centromeres represent 20% of chromosome lengths. This indicates that although CentIER correctly identifies centromeres, it underestimates their size for this species, since Huang et al. (2016) estimated an average of 34% of chromosomes occupied by centromeres in *S. invicta*. In *F. selysi*, the overlap between CentIER predicted centromeres and satellite DNA matches is lower than for *S. invicta*, reflecting the difference in unique satellite DNA sequences identified between the two species and used in the blast approach, only five in *F. selysi* versus 10,000 in *S. invicta*.

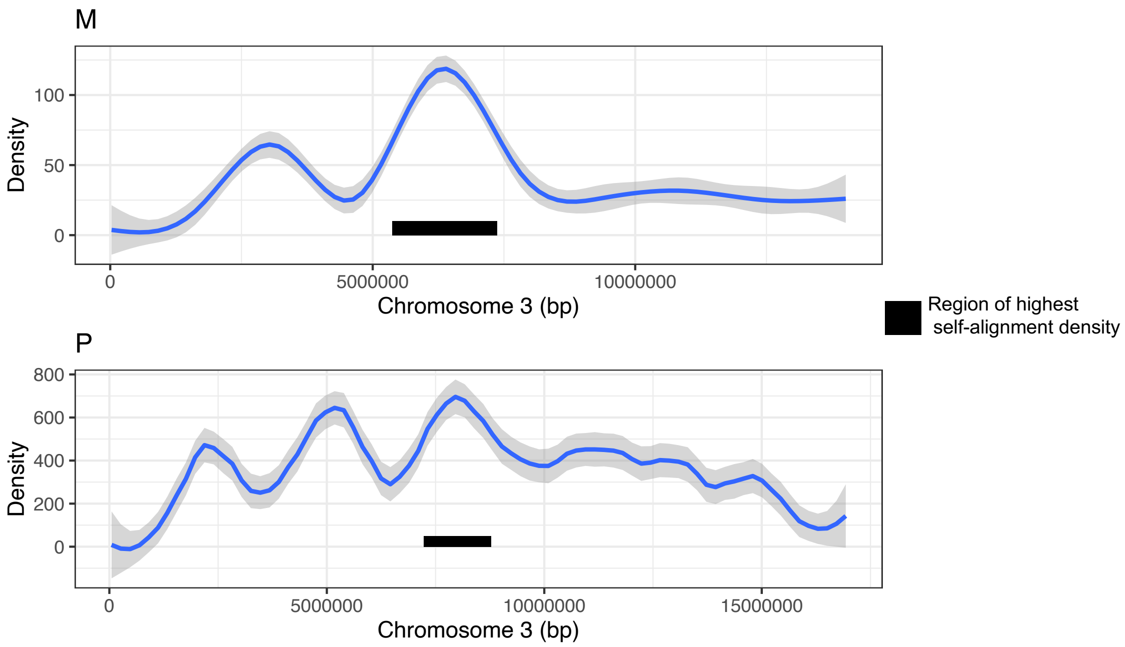

Figure S14. Density of self-alignment across 100kb overlapping windows (step=25kb) in chromosomes 3 from the M genome assembly (top panel) and P genome assembly (bottom panel). Regions of highest self-alignment density were defined as the longest stretches of consecutive windows (>50) with more than 20% of self-alignment. The region of highest self-alignment density ranges from position 5,375,000 to 7375000 in the *M* haplotype, and 7,225,000 to 8,775,000 bp in the *P* haplotype.

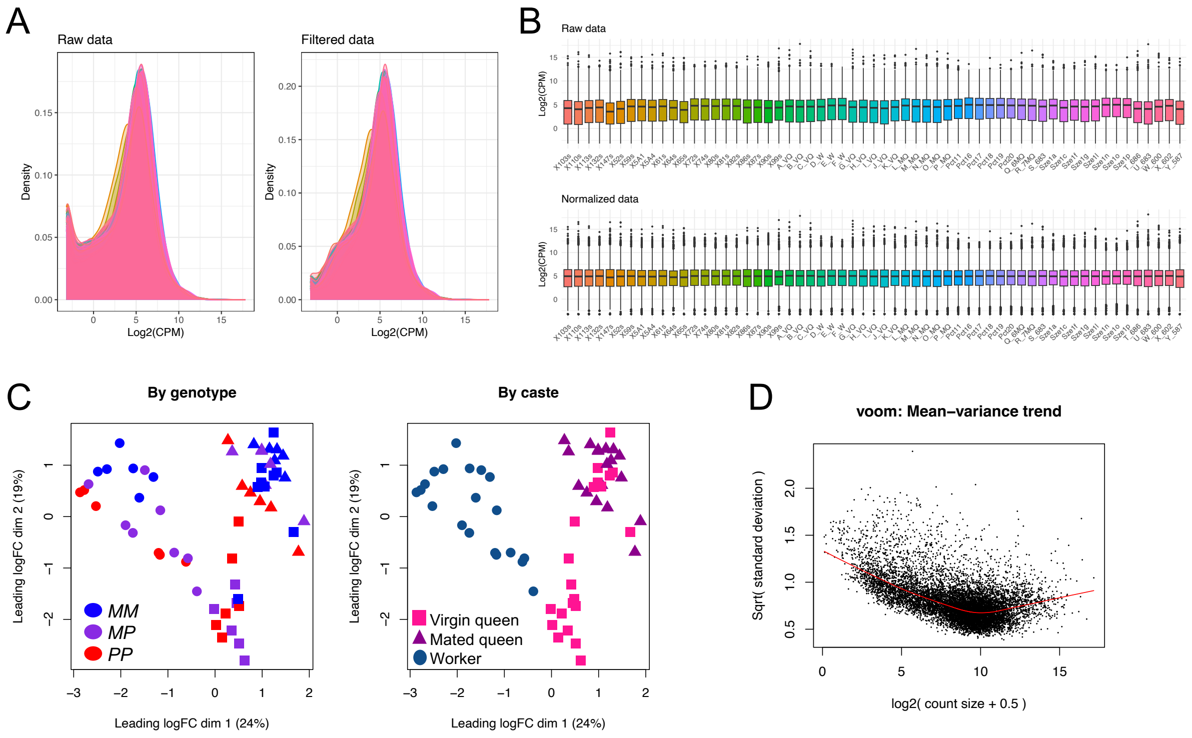

Figure S15. Quality control and exploratory analysis of virgin queen, mated queen and worker RNA-seq data. (A) Density plot of expression before and after filtering for genes with CPM>1 (count per million) in at least six samples. Each curve represents a single sample. Higher density at low CPM values indicates the presence of lowly expressed genes. The filtering process reduces noise by eliminating genes with insufficient counts. (B) Distribution of log2-transformed CPM values before and after normalization with trimmed mean of M values (TMM) method. (C) Multidimensional scaling (MDS) of normalized expression data. Samples are grouped based on genotype and caste. (E) Mean-variance trend from voom transformation (limma package). The red trend line indicates the modeled variance-mean relationship, demonstrating effective variance stabilization after voom transformation.
